# Traditional Uses, Phytochemistry, Pharmacology, and Quality Control of Dendrobium *officinale* Kimura et. Migo

**DOI:** 10.3389/fphar.2021.726528

**Published:** 2021-08-06

**Authors:** Wenhua Chen, Jiemiao Lu, Jiahao Zhang, Jianjun Wu, Lilong Yu, Luping Qin, Bo Zhu

**Affiliations:** School of Pharmaceutical Sciences, Zhejiang Chinese Medical University, Hangzhou, China

**Keywords:** *Dendrobium officinale*, phytochemistry, pharmacology, quality control, traditional use

## Abstract

*Dendrobium officinale*, a well-known plant used as a medicinal and food homologous product, has been reported to contain various bioactive components, such as polysaccharides, bibenzyls, phenanthrenes, and flavonoids. It is also widely used as a traditional medicine to strengthen “Yin”, nourish heart, tonify five viscera, remove arthralgia, relieve fatigue, thicken stomach, lighten body, and prolong life span. These traditional applications are in consistent with modern pharmacological studies, which have demonstrated that *D. officinale* exhibits various biological functions, such as cardioprotective, anti-tumor, gastrointestinal protective, anti-diabetes, immunomodulatory, anti-aging, and anti-osteoporosis effects. In this review, we summarize the research progress of *D. officinale* from November 2016 to May 2021 and aim to better understand the botany, traditional use, phytochemistry, and pharmacology of *D. officinale*, as well as its quality control and safety. This work presents the development status of *D. officinale*, analyzes gaps in the current research on *D. officinale*, and raises the corresponding solutions to provide references and potential directions for further studies of *D. officinale*.

## Introduction

The genus *Dendrobium* (Orchidaceae) includes more than 1,000 species (Teixeira and Ng, 2017). Among them, *Dendrobium officinale* Kimura et. Migo is a precious medicinal plant recorded in Chinese Pharmacopoeia, which is widely used as a traditional Chinese medicine (Editorial Board of China Pharmacopoeia Committee, 2020). Moreover, there is a synonym of “*Dendrobium catenatum* Lindl*.*” in the plant list (http://ipni.org/urn:lsid:ipni.org:names:628140-1). It is well known that *D. officinale* is distributed in several countries around the world, such as the United States, Japan, and Australia. In particular, *D. officinale* exhibits a broader distribution in the different regions of China, including Anhui, Zhejiang, Hunan, Fujian, Guangxi, Sichuan, and Yunnan Provinces (Guo et al., 2020a). Due to the overexploitation and depletion of its wild plant resources, it has been considered as a secondary endangered plant in the “China Plant Red Data Book” (Fu, 1992). On the other hand, the artificial cultivation technology of *D. officinale* has made a significant breakthrough (Cheng Y et al., 2019). However, the existing cultivation resources of *D. officinale* are mixed, which results in the uneven product quality and the unsound evaluation system, greatly affecting the practical and reasonable development and utilization of *D. officinale*.

Pharmacological results published in the literature have revealed multiple promising bioactivities of *D. officinale*, including cardioprotective (Xiao et al., 2018), anti-tumor (Guo et al., 2019), gastrointestinal protective (Liu et al., 2020), anti-diabetes (Zeng et al., 2020), immunomodulatory (Huang et al., 2018), anti-aging (Liang et al., 2017), and anti-osteoporosis (Wang et al., 2018) effects. Besides, it is usually prescribed as one of the ingredients of herbal formula, such as “Tin maple crystal” (Tiepifengdoujing: *D. officinale* and *American ginseng*), for regulating the gastrointestinal tract in traditional and contemporary clinical practice (Qin et al., 2019). According to currently available phytochemical investigations, the bioactive chemical components of *D. officinale* mainly consist of polysaccharides (Zhao et al., 2019), bibenzyls (Zhao et al., 2020), phenanthrenes (Liu et al., 2019), flavonoids (Xing et al., 2018), and alkaloids (Chu et al., 2019). In contrast, according to the literature, only few of these compounds have been evaluated in bioactivity assays (Zhao et al., 2018a; Liu et al., 2018; Xing et al., 2018; Chu et al., 2019; Liu et al., 2019; Lee et al., 2020; Zhao et al., 2020). Additionally, although *D. officinale* is utilized as an effective matter to treat various diseases, its quality control and safety have yet to be defined.

The present review provides an up-to-date and comprehensive literature analysis of *D. officinale* on the basis of botany, traditional applications, phytochemistry, pharmacology, quality control, and safety, which may provide new insights into the development and applications of *D. officinale* as a novel therapeutic agent for the prevention and treatment of diseases.

## Materials and Methods

All the information available about *D. officinale* was obtained from scientific databases, including Web of Science, PubMed, Google Scholar, Baidu Scholar, Springer, Sci Finder, and ScienceDirect, CNKI, from November 2016 to May 2021, and classic books of Chinese herbal medicines. The keywords included *Dendrobium officinale*, *Dendrobium catenatum*, ethnopharmacology, phytochemistry, biological activity, pharmacology, clinic, traditional uses, safety, quality control, toxicology, and other related words. In this paper, *D. catenatum* was also written as *D. officinale*. However, the other species in *Dendrobium* genera were excluded.

## Botany

**FIGURE 1:**
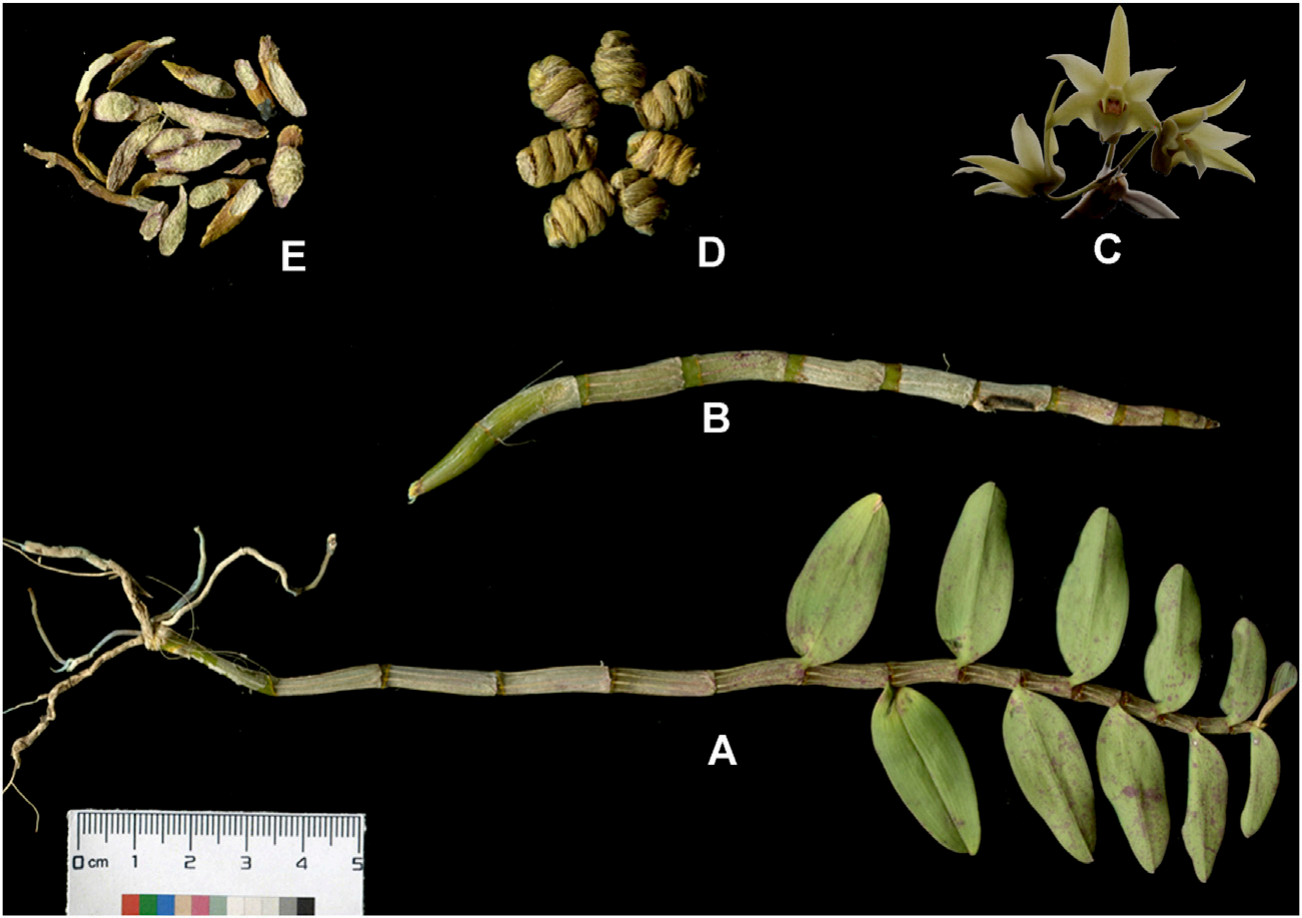
The whole plant **(A)**, fresh strips **(B)**, flowers **(C)**, commercial product named *Tiepifengdou* **(D)**, and decoction pieces **(E)** of *Dendrobium officinale* Kimura et. Migo.

According to the Chinese Pharmacopoeia (Editorial Board of China Pharmacopoeia Committee, 2020), *D. officinale* can be twisted into a spring or spiral shape, which can be further heated and dried to prepare *Tiepifengdou*. *D. officinale* typically has 2–6 whorls with a length of 3–12 cm and a diameter of 0.2–0.4 cm after straightening. The surface color of *D. officinale* is yellow-green or light golden yellow, with fine longitudinal wrinkles, obvious nodes. Moreover, sometimes residual gray-white leaf sheaths can be seen on the nodes. The short fibrous roots remaining at the stem base can be observed at one end (Flora of China Editorial Committee, 2009). *D. officinale* is solid and fragile with a flat section while showing gray-white to gray-green color and slightly horny (Figure 1).

“Flora of China” records: *D. officinale* grows upright to a height of 9–35 cm and a thickness of 2–4 mm and is usually cylindrical. The stem is vertical without branches while having many nodes above the middle and 3–5 leaves alternate. The leaves are biseriate, papery, and oblong-lanceolate. The apex is obtuse and slightly hooked. The leaf-sheaths often exhibit purple spots. Its inflorescence racemes usually come from the upper part of old stems, with deciduous leaves and 2–3 flowers. The inflorescence axis is bent back and is 2–4 cm long. Sepals and petals show yellow-green color and comparable sizes, which are usually end-rounded and have five veins (Flora of China Editorial Committee, 2009). The flowering period is from March to June (Zhao et al., 2018a; Liu et al., 2018).

*D. officinale* is well-known for its nutritional and medicinal value (Flora of China Editorial Committee, 2009), which often grows in semi-humid mountain rocks over 1,600 m and is widely distributed in Anhui, Zhejiang, Fujian, Guangxi, Sichuan, Yunnan Provinces in China. Moreover, *D. officinale* displays a broad geographical distribution in other countries or regions, including India, Australia, Japan, and United States (Guo et al., 2020a).

## Traditional Uses

*D. officinale* is mainly used as traditional medicine and healthy food in China. *D. officinale* was initially recorded in “Shennong’s Herbal Classic” (《神农本草经》, Dong Han Dynasty, A.D. 25–220), which has the ability to strengthen “Yin”, tonify five viscera, nourish heart, remove arthralgia, relieve fatigue, thicken stomach, lighten the body, and prolong life span. In “Variorum of Classic of Herbology” (《本草经集注》, Liang Dynasty, A.D. 502–557), the appearance of *D. officinale* was mentioned to be rough, fine, and solid with a golden color. Moreover, the quality is better if the shape is like grasshopper legs. In “Records of Famous Doctors” (《名医别录》, Weijin Dynasty, A.D. 220–450), *D. officinale* was recorded to be non-toxic and could be widely used to nourish the essence, reinforce kidney, calm the stomach, build muscles, expel evil heat, relieve foot and knee pain, and remove convulsion. In “Theory of Property” (《药性论》, Tang Dynasty, A.D. 618–907), it was stated that *D. officinale* could replenish “Qi” and remove heat. Moreover, Herbal Medicines for Kaibao (《开宝本草》, Song Dynasty, A.D. 960–1,279) also had similar description regarding the medical benefits of *D. officinale* as described above. In addition, it was also recorded in “Amplified Herbology” (《本草衍义》, Song Dynasty, A.D. 960–1,127) that *D. officinale* could treat asthenia heat in the stomach. In “Compendium of Herbology” (《本草纲目》, Ming Dynasty, A.D. 1,552–1,578), *D. officinale* was described as a therapeutic approach for fever, spontaneous sweating, ulcer, and pus. According to the record in “Herbal classics” (《本草经疏》, Ming Dynasty, A.D. 1,552–1,578), *D. officinale* shows the function to remove dampness of spleen and stomach. It was reported that *D. officinale* could treat deficiency of spleen and stomach and relieve internal heat or fever in “Herbal Medicines for Mengquan” (《本草蒙荃》, Ming Dynasty, A.D. 1,552–1,578). In “Essential Herbs” (《本草备要》, Qing Dynasty, A.D. 1,636–1,912), it was documented that *D. officinale* could invigorate spleen and kidney. Similarly, in “Herbal Medicines for Qiuzhen” (《本草求真》, Qing Dynasty, A.D. 1,636–1,912), it was registered that *D. officinale* has the ability to enter the spleen to remove deficiency heat and kidney astringency. Additionally, it was recorded that *D. officinale* could mitigate stomach deficiency and heat (inflammation) and check for polydipsia in “Match Medica” (《得配本草》, Qing Dynasty, A.D. 1,636–1,912). The summary of the traditional medicinal uses is shown in Table 1.

**TABLE 1:** Summary of traditional medicinal uses of *D. officinale*.

Traditional use	Part used	Ref.
Removing arthralgia, tonifying five viscera, relieving fatigue, strengthening Yin, thickening stomach, lightening body, and prolonging life	Stem	Shennong’s Herbal Classic (《神农本草经》) (Dong Han Dynasty, A.D. 25–220)
Raw stone, fine and solid, color of gold, and the shape of grasshopper legs is better	Stem	Variorum of Classic of Herbology (《本草经集注》) (Liang Dynasty, A.D. 502–557)
Nourishing the essence, reinforcing kidney, calming stomach, building muscles, expeling evil heat, relieving foot and knee pain, and removing convulsion	Stem	Records of Famous Doctors (《名医别录》) (Weijin Dynasty, A.D. 220–450)
Replenishing Qi and removing heat	Stem	Theory of Property (《药性论》) (Tang Dynasty, A.D. 618–907)
Nourishing the essence, calming stomach, building muscles, expelling skin evil heat, relieving foot and knee pain	Stem	Herbal Medicines for Kaibao (《开宝本草》) (Song Dynasty, A.D. 960–1,279)
Treating asthenia heat in stomach	Stem	Amplified Herbology (《本草衍义》) (Song Dynasty, A.D. 960–1,127)
Treating fever, spontaneous sweating, ulcer, and pus	Stem	Compendium of Herbology (《本草纲目》) (Ming Dynasty, A.D. 1,552–1,578)
Removing dampness of spleen and stomach	Stem	Herbal classics (《本草经疏》) (Ming Dynasty, A.D. 1,552–1,578)
Treating deficiency of spleen and stomach, and relieve internal heat or fever	Stem	Herbal Medicines for Mengquan (《本草蒙荃》) (Ming Dynasty, A.D. 1,552–1,578)
Invigorating spleen and kidney	Stem	Essential Herbs (《本草备要》) (Qing Dynasty, A.D. 1,636–1912)
Entering the spleen to remove deficiency heat and kidney astringency	Stem	Herbal Medicines for Qiuzhen (《本草求真》) (Qing Dynasty, A.D. 1,636–1912)
Removing stomach deficiency heat and check polydipsia	Stem	Match Medica (《得配本草》) (Qing Dynasty, A.D. 1,636–1912)

*D. officinale* has been reported to have effects on benefiting heart and stomach to produce saliva and nourishing “Yin” to clear heat in China Pharmacopoeia Committee, which is mainly utilized in the treatment of fluid injury, deficiency of “Yin” in the heart and stomach, no regression of inflammation after the disease, hyperactivity of heat due to “Yin” deficiency, dark eyes, muscles, and bone weakness in accordance with China Pharmacopoeia Committee (Committee for the Pharmacopoeia of PR China, 2015). On the other hand, *D. officinale* stems can be used as ordinary food (National Health and Family Planning Commission of the People’s Republic of China, 2013). In traditional Chinese therapies, *D. officinale* is often combined with other Chinese herbal medicines, including *Adenophora stricta*, *Ophiopogon japonicus*, *Paeonia lactiflor*a, *Astragalus membranaceus* and *Polygonatum odoratum*. In recent years, pharmacological research mainly focused on the cardioprotective (Xiao et al., 2018), anti-tumor (Guo et al., 2019), gastrointestinal protective (Liu et al., 2020), and anti-diabetes (Zeng et al., 2020) effects of *D. officinale*. Therefore, it is encouraged to combine the traditional pharmacological actions of *D. officinale* and advanced research technologies in order to explore the potential mechanisms and establish the foundation for its clinical applications in the future.

## Phytochemistry

**FIGURE 2:**
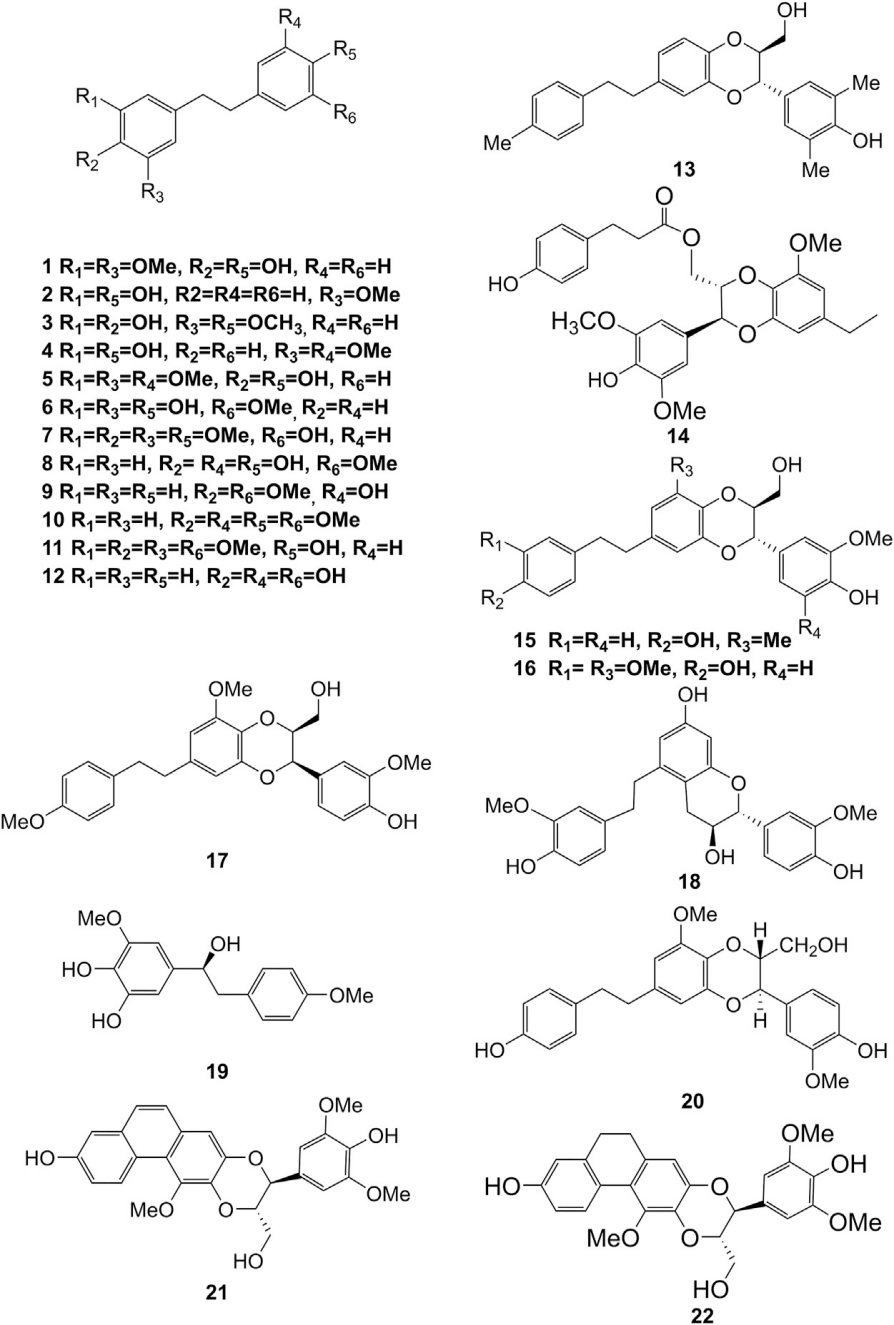
Structures of bibenzyls (**1–22**) isolated from *D. officinale*.

**FIGURE 3:**
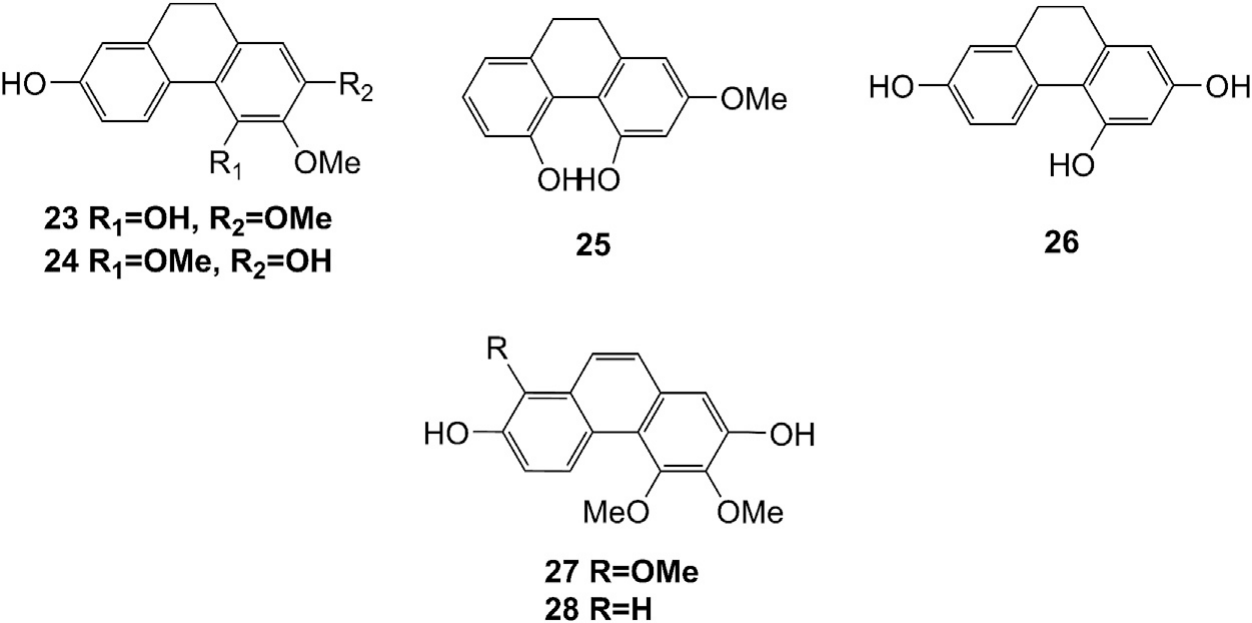
Structures of phenanthrenes (**23–28**) isolated from *D. officinale*.

**FIGURE 4:**
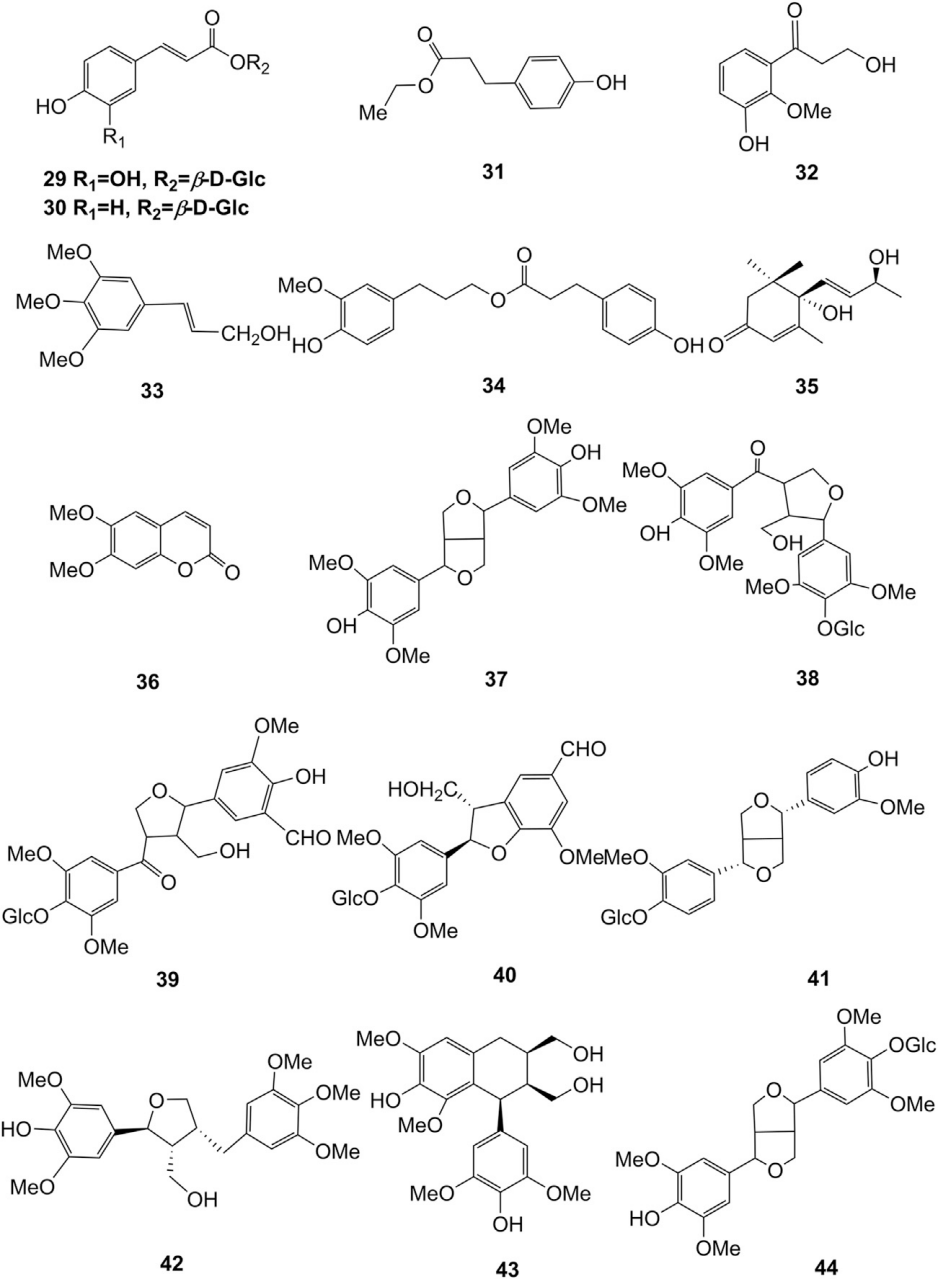
Structures of phenylpropanoids (**29–44**) isolated from *D. officinale*.

**FIGURE 5:**
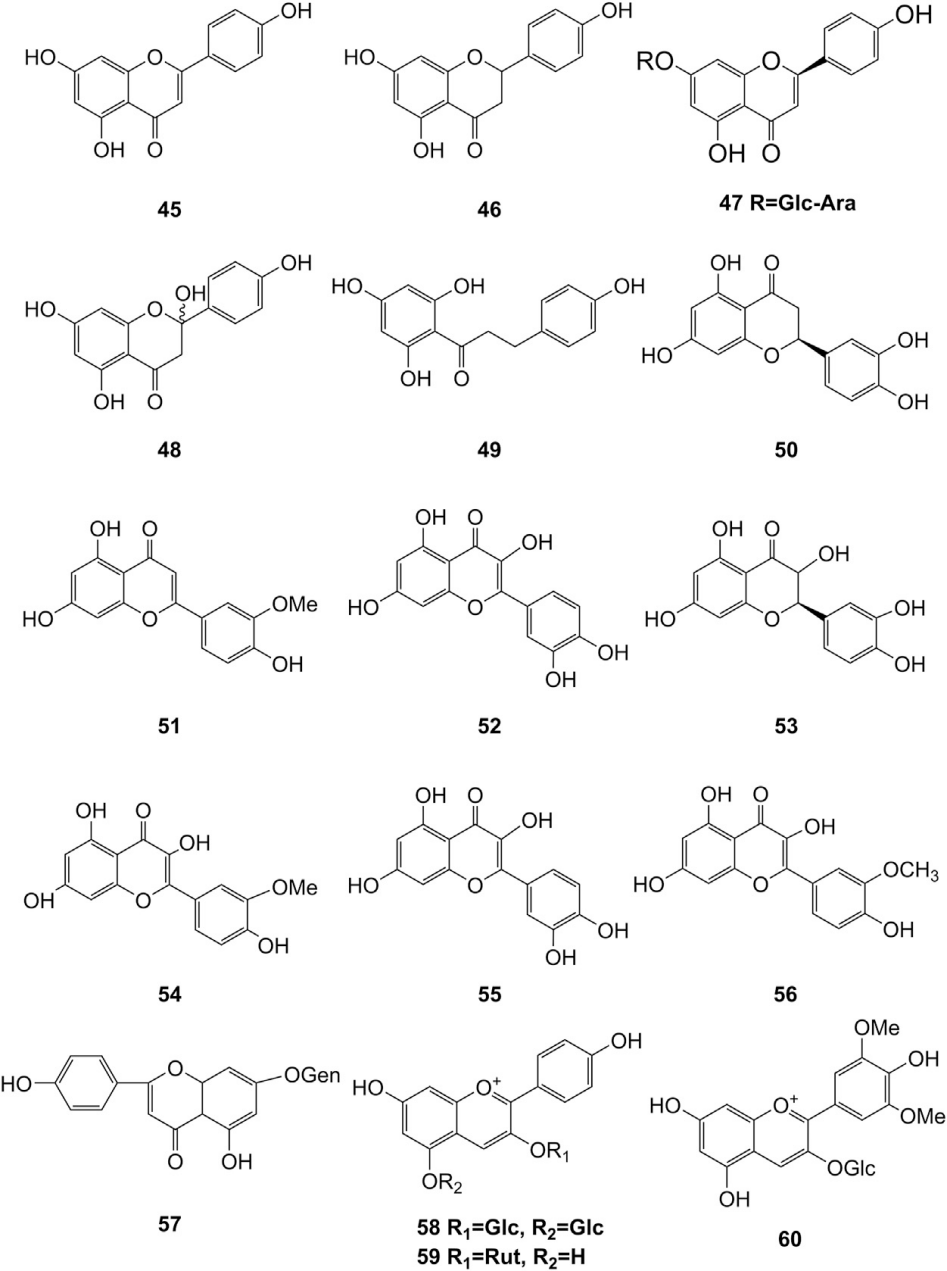
Structures of flavonoids (**45–60**) isolated from *D. officinale*.

**FIGURE 6:**
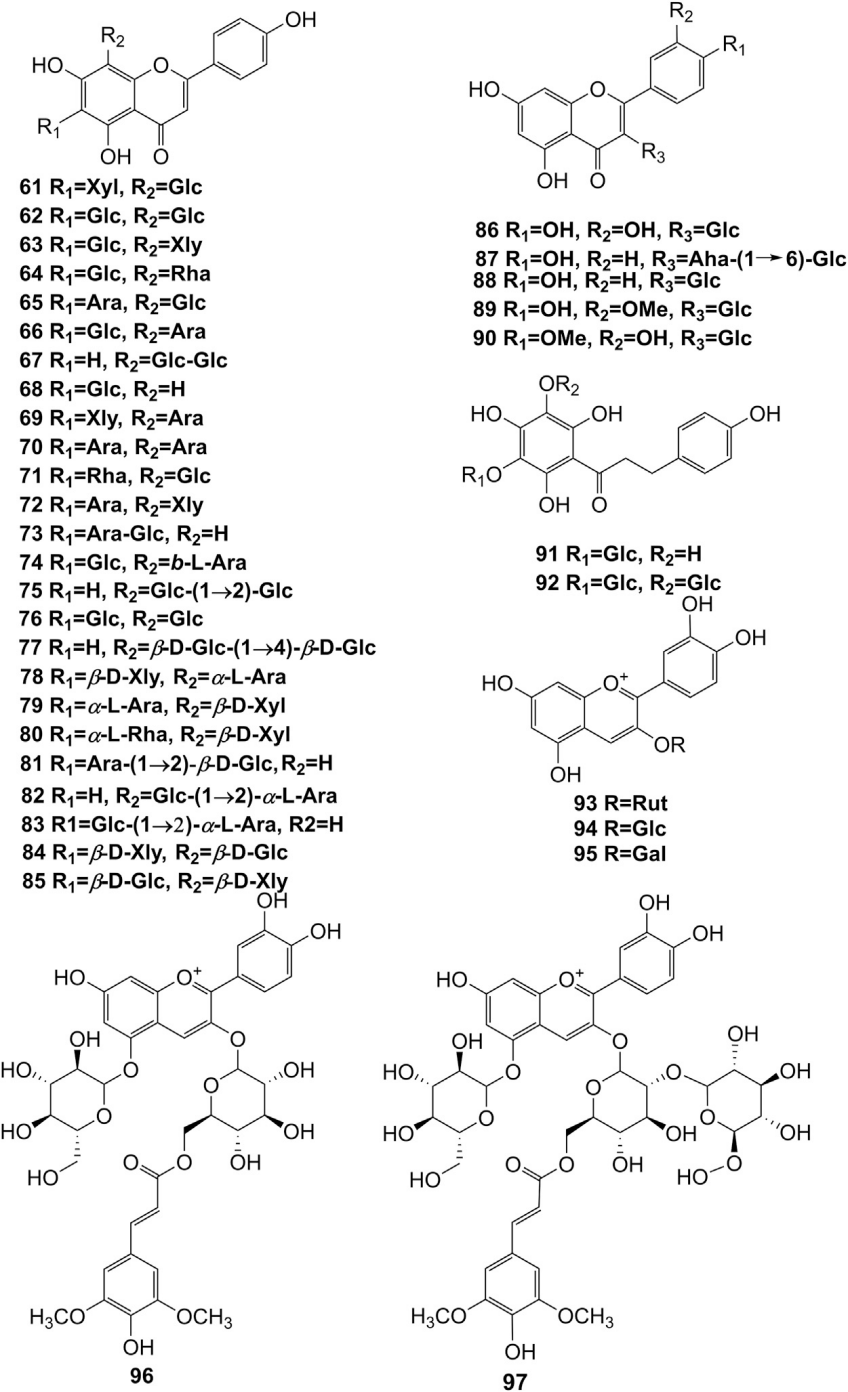
Structures of flavonoids (**61–97**) isolated from *D. officinale*.

**FIGURE 7:**
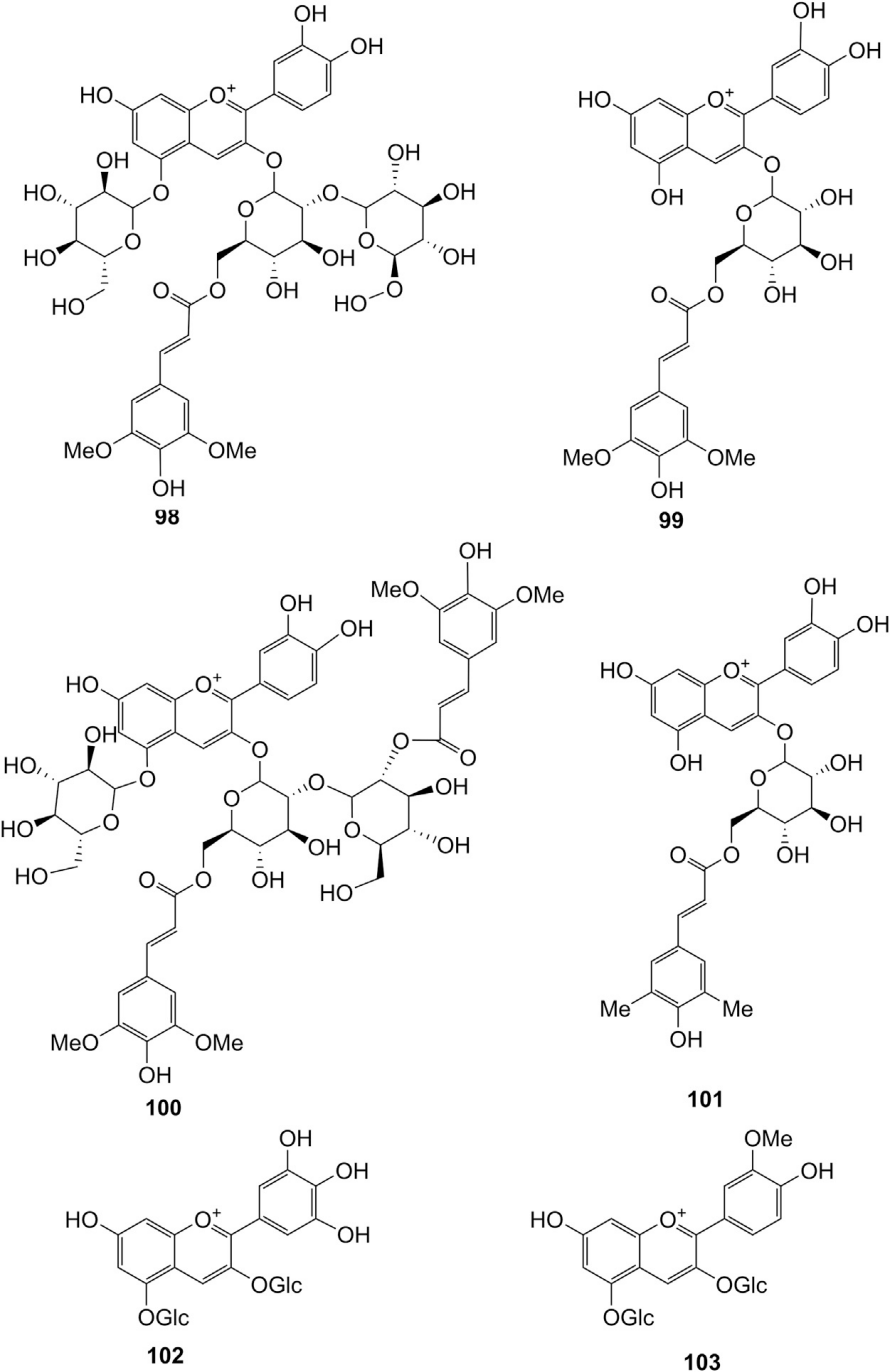
Structures of flavonoids (**98–103**) isolated from *D. officinale*.

**FIGURE 8:**
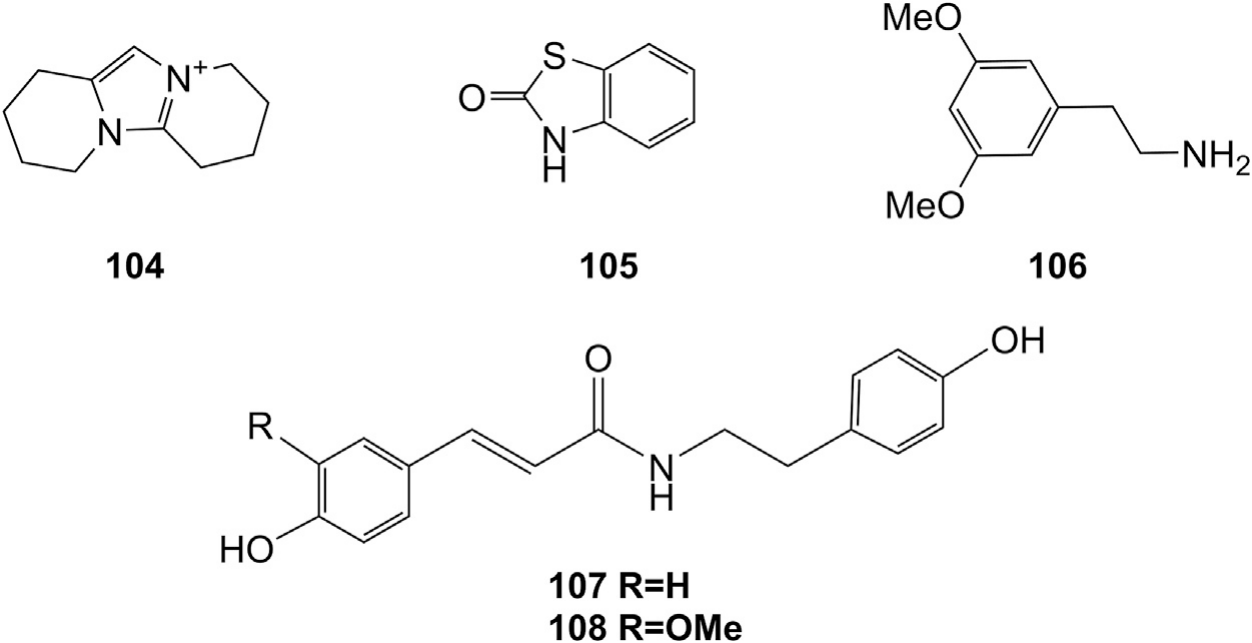
Structures of alkaloids (**104–108**) isolated from *D. officinale*.

Extensive phytochemistry studies have demonstrated that *D. officinale* mainly contains polysaccharides, bibenzyls (**1–22**), phenanthrenes (**23–28**), phenylpropanoids (**29–44**), flavonoids (**45–103**), alkaloids (**104–108**), acids (**109–116**), and others (**117–126**). Among them, polysaccharides, bibenzyls, and flavonoids are considered the main bioactive compounds responsible for various pharmacological properties and therapeutic efficacy of *D. officinale*. All compounds identified in *D. officinale* are summarized and shown in Table 2, and the corresponding structures are shown in Figures 2–8.

**TABLE 2:** The main compounds isolated from *D. officinale*.

Class	No	Name	Formula	Plant parts	Ref.
Bibenzyls	1	4,4′-Dihydroxy-3,5-dimethoxybibenzyl	C_16_H_18_O_4_	Leaves	Ren et al. (2020a); Ren et al. (2020b)
	2	3,4′-Dihydroxy-5-methoxybibenzyl	C_15_H_16_O_3_	Stems	Liu et al. (2018)
	3	3,4-Dihydroxy-4′,5-dimethoxybibenzyl	C_16_H_18_O_4_	Leaves	Ren et al. (2020a); Ren et al. (2020b)
	4	Gigantol	C_16_H_18_O_4_	Stems, Leaves	He et al. (2020); Ren et al. (2020b)
	5	Moscatilin	C_17_H_20_O_5_	Leaves	Ren et al. (2020a); Ren et al. (2020b)
	6	Tristin	C_15_H_16_O_4_	Stems	He et al. (2020)
	7	Erianin	C_18_H_22_O_5_	Stems	Peng et al. (2020)
	8	3,4,4′-Trihydroxy-5-methoxybibenzyl	C_15_H_16_O_4_	Stems	Chu et al. (2019)
	9	3-Hydroxy-4′,5-dimethoxybibenzy	C_16_H_18_O_3_	Stems	Chu et al. (2019)
	10	Amoenylin	C_17_H_20_O_4_	Stems	Chu et al. (2019)
	11	Dendrophenol	C_17_H_20_O_5_	Stems	Peng et al. (2020)
	12	Dihydroresveratrol	C_14_H_14_O_3_	Stems	Liu et al. (2018)
	13	Dendrocandin B	C_27_H30O_8_	Leaves	Ren et al. (2020a); Ren et al. (2020b)
	14	Denofficin	C_36_H_38_O_10_	Leaves	Ren et al. (2020b)
	15	Dendrocandin U	C_26_H_28_O_8_	Stems, Leaves	Liu et al. (2018); Ren et al. (2020a); Ren et al. (2020b)
	16	Dendrocandin W	C_26_H_28_O_8_	Stems	Zhu et al. (2020)
	17	Dendrocandin V	C_26_H_28_O_7_	Stems	Zhu et al. (2020)
	18	Trigonopol B	C_25_H_26_O_7_	Stems	Zhu et al. (2020)
	19	3,4, α-Trihydroxy-5,4′-dimethoxybibenzyl	C_16_H_18_O_5_	Laves	Ren et al. (2020a); Ren et al. (2020b)
	20	Dendrocandin N	C_25_H_26_O_7_	Stems	Zhu et al. (2020)
	21	Dendrocandin P1	C_26_H_24_O_8_	Stems	He et al. (2020)
	22	Dendrocandin P2	C_26_H_26_O_8_	Stems	He et al. (2020)
Phenanthrenes	23	Ephemeranthol A	C_16_H_16_O_4_	Stems	Cui et al. (2019); He et al. (2020)
	24	Erianthridin	C_16_H_16_O_4_	Stems	Cui et al. (2019)
	25	Orchinol	C_15_H_14_O_3_	Stems	He et al. (2020)
	26	2,4,7-Trihydroxy-9, 10-dihydrophenanthrene	C_14_H_12_O_3_	Stems	He et al. (2020)
	27	Confusarin	C_17_H_16_O_5_	Stems	Cui et al. (2019); He et al. (2020)
	28	2,7-Dihydroxy-3, 4-dimethoxyphenanthrene	C_16_H_14_O_4_	Stems	Cui et al. (2019)
Penylpropanoids	29	1-O-Caffeoyl-β-D-glucoside	C_15_H_18_O_9_	Flowers	Zhang et al. (2019)
	30	1-O-*p*-Coumaroyl-β-D-glucoside	C_15_H_18_O_8_	Flowers	Zhang et al. (2019)
	31	ethyl *p*-Hydroxyhydrocinnamate	C_11_H_14_O_3_	Leaves	Ren et al. (2020a)
	32	ω-Hydroxypropioguaiacone	C_10_H_12_O_4_	Stems	Zhu et al. (2020)
	33	trans-3,4,5-Trimethoxyl-cinnamyl alcohol	C_12_H_16_O_4_	Stems	Cui et al. (2019)
	34	Dihydroconiferyldihydro-*p*-coumarate	C_19_H_22_O_5_	Stems	Ye et al. (2017)
	35	6-Hydroxy-3-oxo-α-ionol	C_13_H_20_O_3_	Stems	Zhu et al. (2020)
	36	Scoparone	C_11_H_10_O_4_	Stems	Peng et al. (2020)
	37	Syringaresinol	C_22_H_26_O_8_	Stems, Leaves	Ye et al. (2017); Ren et al. (2020a)
	38	Magnolenin C	C_28_H_36_O_14_	Stems	Cui et al. (2019)
	39	Officinalioside	C_28_H_34_O_14_	Stems	Cui et al. (2019)
	40	Moellenoside A	C_26_H_32_O_11_	Stems	Cui et al. (2019)
	41	Pinoresinol-4-O-β-D-glucopyranoside	C_28_H_36_O_14_	Stems	Cui et al. (2019)
	42	5, 5′-Dimethoxy-lariciresinol	C_23_H_30_O_8_	Stems	He et al. (2020)
	43	Lyoniresinol	C_22_H_28_O_8_	Stems	He et al. (2020)
	44	(+)-Syringaresinol-4′-O*-*β-D-glucopyranoside	C_28_H_36_O_13_	Stems	Chen et al. (2018)
Flavonoids	45	Apigenin	C_15_H_10_O_5_	Roots, Stems, Leaves	Yu et al. (2018); Ren et al. (2020c)
	46	Naringenin	C_15_H_12_O_5_	Stems	Ye et al. (2017); Peng et al. (2020)
	47	Naringin	C_27_H_32_O_14_	Stems	Zhou et al. (2018)
	48	2-Hydroxynaringenin	C_15_H_12_O_6_	Roots, Stems, Laves	Ren et al. (2020c)
	49	Phloretin	C_15_H_14_O_5_	Roots, Stems	Ren et al. (2020c)
	50	Eriodictyol	C_15_H_12_O_6_	Stems	Lv et al. (2017)
	51	Chrysoeriol	C_16_H_12_O_6_	Stems	Lv et al. (2017)
	52	Quercetin	C_15_H_10_O_7_	Stems	Lv et al. (2017)
	53	Taxifolin	C_15_H_12_O_7_	Stems	Lv et al. (2017)
	54	Isorhamnetin	C_16_H_12_O_7_	Stems	Lv et al. (2017)
	55	Rutin	C_27_H_30_O_16_	Stems, Leaves, Flowers	Ye et al. (2017); Zhou et al. (2018); Ren et al. (2020a); Zhang et al. (2019)
	56	Cosmosiin	C_21_H_20_O_10_	Flowers	Ren et al. (2020c)
	57	Genistin 7-O-gentiobioside	C_27_H_30_O_15_	Stems	Yu et al. (2018)
	58	Pelargonidin 3,5-O-diglucoside	C_27_H_31_O_15_	Stems	Yu et al. (2018)
	59	Pelargonidin 3-O-rutinoside	C_27_H_31_O_14_	Stems	Yu et al. (2018)
	60	Malvidin 3-O-glucosid	C_23_H_25_O_12_	Stems	Yu et al. (2018)
	61	Vicenin I	C_26_H_28_O_14_	Roots, Stems, Leaves, Flowers	Ye et al. (2017); Ren et al. (2020c); Zhang et al. (2017b)
	62	Vicenin II	C_27_H_30_O_15_	Roots, Stems, Leaves, Flowers	Lv et al. (2017); Zhang et al. (2017b); Luo et al. (2019)
	63	Vicenin III	C_26_H_28_O_14_	Stems	Lei et al. (2018)
	64	Violanthin	C_27_H_30_O_14_	Stems	(Ye et al. 2017; Lei et al. 2018; Zhou et al. 2018)
	65	Isoschaftoside	C_26_H_28_O_14_	Stems, Flowers	Ye et al. (2017); Zhou et al. (2018); Zhang et al. (2019)
	66	Schaftoside	C_26_H_28_O_14_	Stems, Flowers	Ye et al. (2017); Zhou et al. (2018); Zhang et al. (2019)
	67	Vitexin-2″-O-β-D-glucopyranoside	C_26_H_27_O_14_	Stems	Ye et al. (2017)
	68	Isovitexin apigenin-6-C-glucoside	C_21_H_20_O_10_	Stems	Ren et al. (2020c)
	69	Apigenin-6-C-β-D-xyloside-8-C-β-D-arabinoside	C_25_H_26_O_13_	Stems	Ye et al. (2017)
	70	Apigenin-6,8-di-C-α-L-arabinoside	C_25_H_26_O_13_	Stems, Flowers	Ye et al. (2017); Zhang et al. (2019)
	71	Isoviolanthin	C_27_H_30_O_14_	Stems, Leaves	Ye et al. (2017); Zhang et al. (2019)
	72	Apigenin-6-C-α-L-arabinoside-8-C-β-D-xyloside	C_25_H_26_O_13_	Stems	Ye et al. (2017)
	73	Apigenin-6-C-(2″-O-β-Dglucopyranoside)-α-L-arabinoside	C_26_H_27_O_13_	Stems	Ye et al. (2017)
	74	Neoschaftoside	C_26_H_28_O_14_	Flowers	Zhang et al. (2019)
	75	Vitexin-2″-O-glucoside	C_27_H_30_O_15_	Flowers	Zhang et al. (2019)
	76	Apigenin-6,8-di-C-β-D-glucoside	C_27_H_30_O_15_	Flowers	Zhou et al. (2018); Zhang et al. (2019)
	77	Apigenin-8-C-β-D-glucosyl-(1→4)-O-β-D-glucoside	C_27_H_30_O_15_	Flowers	Zhang et al. (2019)
	78	Apigenin-6-C-β-D-xyloside-8-C-α-L-arabinoside	C_25_H_26_O_13_	Flowers	Zhang et al. (2019)
	79	Apigenin-6-C-α-L-arabinoside-8-C-β-D-xyloside	C_25_H_26_O_13_	Flowers	Zhang et al. (2019)
	80	Apigenin-6-C-α-L-rhamnoside-8-C-β-D-xyloside	C_26_H_28_O_13_	Flowers	Zhang et al. (2019)
	81	Apigenin-6-C-arabinosyl-2″-O-β-D-glucoside	C_26_H_28_O_14_	Flowers	Zhang et al. (2019)
	82	Apigenin-8-C-glucosyl-(1→2)-α-L-arabinoside	C_26_H_28_O_14_	Flowers	Zhang et al. (2019)
	83	Apigenin 6-C-glucosyl-(1→2)-α-L- arabinoside	C_26_H_28_O_14_	Leaves	Zhang et al. (2017b)
	84	Apigenin-6-C-β-D-xyloside-8-C-β-D-glucoside	C_26_H_28_O_15_	Leaves	Zhou et al. (2018)
	85	Apigenin-6-C-β-D-glucoside-8-C-β-D-xyloside	C_26_H_28_O_15_	Leaves	Zhou et al. (2018)
	86	Isoquercitrin	C_21_H_20_O_12_	Flowers	Zhang et al. (2019)
	87	Kaempferol-3-O-α-L-rutinoside	C_27_H_30_O_15_	Flowers	Zhang et al. (2019)
	88	Kaempferol-3-O-β-D-glucoside	C_21_H_20_O_11_	Flowers	Zhang et al. (2019)
	89	Isorhamnetin-3-O-β-D-glucoside	C_22_H_22_O_12_	Flowers	Zhang et al. (2019)
	90	Tamarixin	C_22_H_22_O_12_	Flowers	Zhang et al. (2019)
	91	Nothofagin Glc	C_21_H_24_O_10_	Flowers	Ren et al. (2020c)
	92	3′,5′-Di-C-glucosylphloretin	C_27_H_34_O_15_	Stems	Ren et al. (2020c)
	93	Cyanidin 3-O-rutinoside	C_27_H_31_O_15_	Stems	Ren et al. (2020d)
	94	Cyanidin 3-O-glucoside	C_21_H_21_O_11_	Stems	Yu et al. (2018)
	95	Cyanidin 3-O-galactoside	C_21_H_21_O_11_	Stems	Yu et al. (2018)
	96	Cyanidin 3-[6-(sinapoyl) glucoside]-5-glucoside	C_38_H_41_O_20_	Stems	Ren et al. (2020d)
	97	Cyanidin 3-[2-(glucosyl)-6-(sinapoyl) glucoside]-5-glucoside	C_44_H_51_O_25_	Stems	Ren et al. (2020d)
	98	Cyanidin 3-[6-sinapoyl-2-O-(2-(sinapoyl) glucosyl)-glucoside]	C_49_H_51_O_24_	Stems	Ren et al. (2020d)
	99	Cyanidin 3-[6-sinapoyl-2-O-(2-(sinapoyl) glucosyl)- glucoside]-5-glucoside	C_55_H_61_O_29_	Stems	Ren et al. (2020d)
	100	Cyanidin 3-[6-(sinapoyl)glucoside]	C_32_H_31_O_15_	Stems	Ren et al. (2020d)
	101	Delphinidin 3-glucoside-7, 3′-di-[6-(sinapoyl) glucoside]	C_55_H_61_O_30_	Stems	Ren et al. (2020d)
	102	Delphinidin 3,5-O-diglucoside	C_27_H_31_O_17_	Stems	Yu et al. (2018)
	103	Peonidin 3,5-O-diglucoside	C_28_H_33_O_16_	Stems	Yu et al. (2018)
Alkaloids	104	Anosmine	C_11_H_17_N_2_	Stems	Chen et al. (2018)
	105	2-Benzothiazolol	C_7_H_5_NOS	Leaves	Ren et al. (2020a)
	106	3,5-Dimethoxyphenethylamines	C_10_H_15_NO_2_	Stems	Zhu et al. (2020)
	107	N-*p*-coumaroyltyramine	C_17_H_17_NO_3_	Stems	Chu et al. (2019)
	108	N-*trans*-*p*-feruloyltyramine	C_18_H_19_NO_4_	Stems	Chu et al. (2019)
Acids	109	Malic acid	C_4_H_6_O_5_	Stems	Chen et al. (2018)
	110	Ferulic acid	C_10_H_10_O_4_	Stems	Ye et al. (2017)
	111	Vanillic acid	C_8_H_8_O_4_	Stems	Ye et al. (2017)
	112	Syringic acid	C_9_H_10_O_5_	Stems	Ye et al. (2017)
	113	Protocatechuic acid	C_7_H_6_O_4_	Leaves	Ren et al. (2020a)
	114	*p*-Hydroxycinnamic acid	C_9_H_8_O_3_	Stems	Ye et al. (2017)
	115	*p*-Hydroxylbenzoic acid	C_7_H_6_O_3_	Leaves	Ren et al. (2020a)
	116	Palmitic acid	C_16_H_32_O_2_	Leaves	Ren et al. (2020a)
Others	117	Flifimdioside A	C_32_H_52_O_13_	Stems	Chen et al. (2018)
	118	Flickinflimoside B	C_31_H_50_O_13_	Stems	Chen et al. (2018)
	119	Loliolide	C_11_H_16_O_3_	Leaves	Ren et al. (2020a)
	120	1-Glycerol linolenate	C_21_H_36_O_4_	Leaves	Ren et al. (2020a)
	121	Densiflorol A	C_16_H_16_O_4_	Leaves	Ren et al. (2020a); Ren et al. (2020b)
	122	2-Butoxyethyl linolenate	C_24_H_42_O_3_	Leaves	Ren et al. (2020a)
	123	Catechol	C_6_H_6_O_2_	Leaves	Ren et al. (2020a)
	124	Octadecadienoic acid-2,3-dihydroxypropyl ester	C_21_H_40_O_4_	Leaves	Ren et al. (2020a)
	125	Stigmast-5-en-3β-ol-7-one	C_29_H_48_O_2_	Stems	Zhu et al. (2020)
	126	Dendrofindlaphenol B	C_27_H_30_O_6_	Stems	Liu et al. (2018)

### Polysaccharides

Polysaccharides are the main medicinal components of *D. officinale*, with a wide range of medicinal properties*.* A large number of polysaccharides have been isolated from *D. officinale*, including DWDOP1, DWDOP2, DWDOP3, FWDOP1, FWDOP2, FWDOP3 (Yu et al., 2018), DOP-50, DOP-60, DOP-70 (Xing et al., 2018), DO (Ma et al., 2018), DOPA-1 (Wei et al., 2018), DOP1-DES, DOP2-DES (Liang J et al., 2018), LDOP-1 (Yang et al., 2020), UDP-1, FLP-1, and FDP-1 (Liang K. L et al., 2018), which are identified by various analytical technologies, such as high performance liquid chromatography (HPLC), high performance gel permeation chromatography (HPAEC), gas chromatography-mass spectrometry (GC-MS), and gel permeation chromatography (GPC). The molecular weights of *D. officinale* polysaccharides range from 30 to 1,415 kDa (Liang J et al., 2018; Ma et al., 2018; Wei et al., 2018; Yu et al., 2018). It has been demonstrated that *D. officinale* polysaccharides are composed of glucose, mannose, galactose, xylose, arabinose, ribose and rhamnose (Tian et al., 2019). Among them, glucomannan is considered to be the main component of *D. officinale* polysaccharides with 1,4-β-D-Man*p* and 1,4-β-D-Glc*p*, comprising acetyl groups in varying degrees and positions with or without branches (Liang et al., 2019). It should be mentioned that a lot of factors may impact the biological activity of polysaccharides, including molecular structure, main chain composition, molecular branching degree, the configuration of the main chain, and chemical modification (Yue et al., 2017; Huang et al., 2020). Importantly, according to the literature, polysaccharides have exhibited various therapeutic potentials, including cardioprotective (Zhang et al., 2017a; Su et al., 2021), anti-tumor (Wei et al., 2018; Zhao et al., 2019), gastrointestinal protective (Ke et al., 2020; Zhang et al., 2020), immunomodulatory (Huang et al., 2018), anti-aging (Liang et al., 2017; Wei et al., 2017), and pulmonary protective effects (Chen et al., 2020).

### Bibenzyls

Bibenzyl components in *D. officinale* have attracted a lot of attention due to their promising anti-tumor properties (He et al., 2020). To date, 19 bibenzyls have been isolated, which were identified predominantly from the stems and leaves of *D. officinale*. The typical parent nucleus structure of bibenzyls presented in the stems of *D. officinale* is two benzene rings, which can be replaced by different substituents. Some bibenzyls (**4**, **5**, and **7**) were found to have significant anti-tumor activity (Tian et al., 2019; Zhao et al., 2020), which attracted further research and development. The chemical structures of these bibenzyls are shown in Figure 2.

### Phenanthrenes

The phenanthrenes are abundantly found in the stems of *D. officinale*, including ephemeranthol A (**23**), erianthridin (**24**), orchinol (**25**), 2, 4, 7-trihydroxy-9, 10-dihydrophenanthrene (**26**), confusarin (**27**), and 2,7-dihydroxy-3,4-dimethoxyphenanthrene (**28**). Notably, orchinol (**25**) possesses anti-tumor activity, which can potentially be used to develop new anti-tumor drugs (Zhao et al., 2018a). The chemical structures of these phenanthrenes are shown in Figure 3.

### Phenylpropanoids

Phenylpropanoid compounds refer to natural organic compounds with one or more C_6_-C_3_ units in the basic parent nucleus, mainly including simple phenylpropanoids, coumarins, and lignans. To date, 16 phenylpropanoids (**29–44**) have been identified in *D. officinale*, including simple phenylpropanoids (**29–35**), coumarin (**36**), and lignans (**37–44**). The chemical structures of these phenylpropanoids are presented in Figure 4.

### Flavonoids

It has been reported that flavonoids belong to a large group of secondary metabolites in *D. officinale*, which possess anti-tumor activity (Xing et al., 2018). Most flavonoids isolated and identified from the roots, stems, leaves, and flowers of *D. officinale* are *C*-glycosides, while the rest of flavonoids are *O*-glycosides. It is well known that the foundational skeletons of flavonoids are apigenin, vitexin, quercetin, and kaempferol. Nowadays, mass spectrometry technologies coupled with liquid chromatography, such (HPLC-ESI-MS) (Ye et al., 2017), ultra-high-performance liquid chromatography (UPLC-ESI-MS/MS) (Zhou et al., 2018), and UPLC-quadrupole time of flight mass spectrometry (UPLC-QTOF-MS) (Yu et al., 2018), have been widely implemented for the identification and quantification of these flavonoid compounds. Interestingly, it is generally believed that glucoside derivatives (anthocyanins) delphinidin 3,5-*O*-diglucoside and cyanidin 3-*O*-glucoside are responsible for the red color stems of *D. officinale* (Yu et al., 2018). In addition, the transcriptome and component metabolism analyses have become the typical approaches to investigate flavonoid bio-synthesis mechanisms in *D. officinale* (Lei et al., 2018). The chemical structures of these flavonoids are shown in Figures 5–7.

### Alkaloids

Alkaloids are a class of nitrogenous organic compounds possessing various biological activities. They are also active constituents of *Dendrobium* plants. It is worth noting that the regulation of genes related to alkaloid biosynthetic pathways through comparative transcriptomic analysis has gradually become a research interest (Jiao et al., 2018). The chemical structures of these alkaloids are shown in Figure 8.

### Organic Acids

**FIGURE 9:**
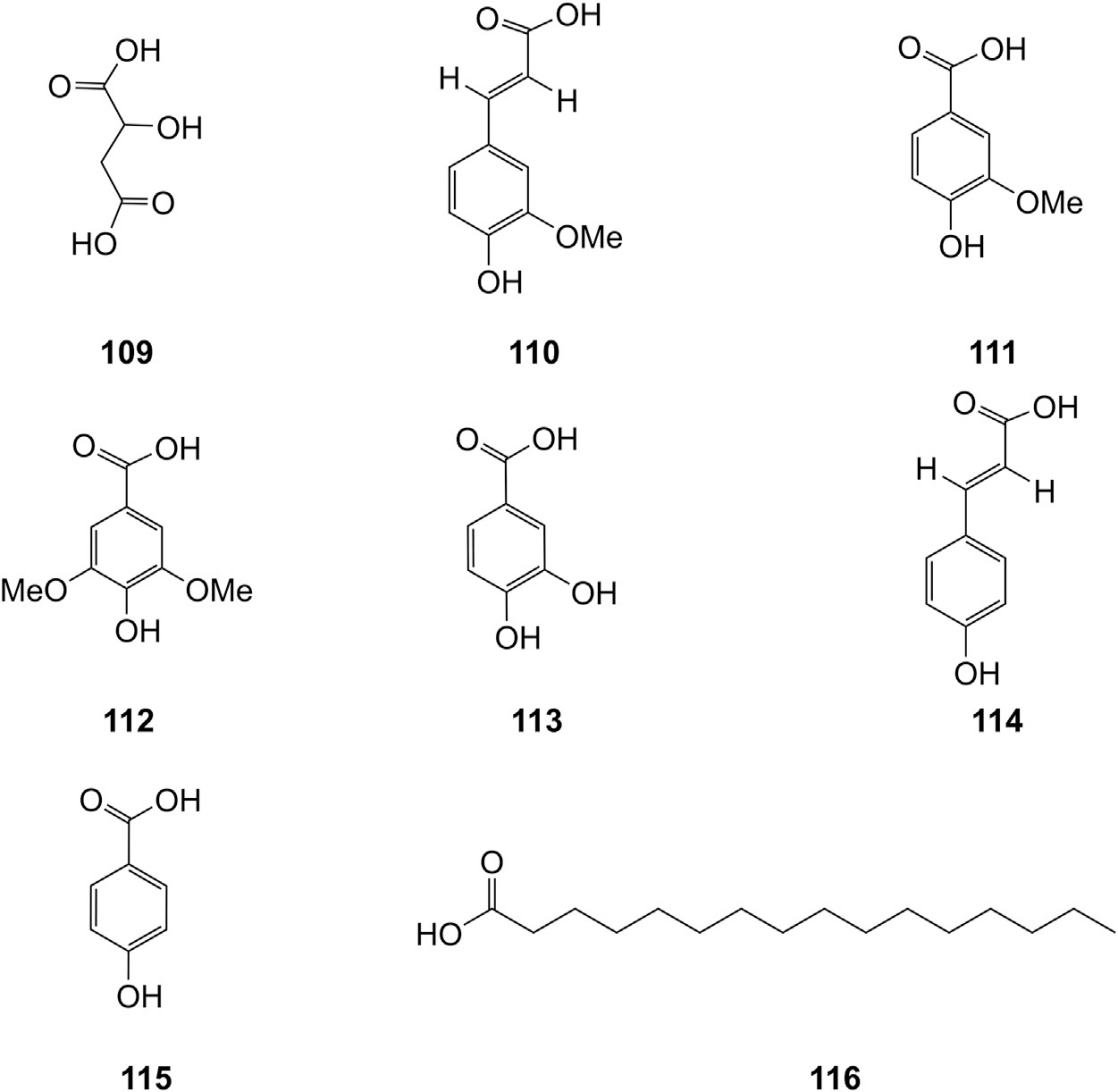
Structures of acids (**109–116**) isolated from *D. officinale*.

Organic acids are a kind of acid organic compounds containing a carboxyl group. Eight types of organic acids have been identified in *D. officinale*. The chemical structures of these organic acids are displayed in Figure 9.

### Other Compounds

**FIGURE 10:**
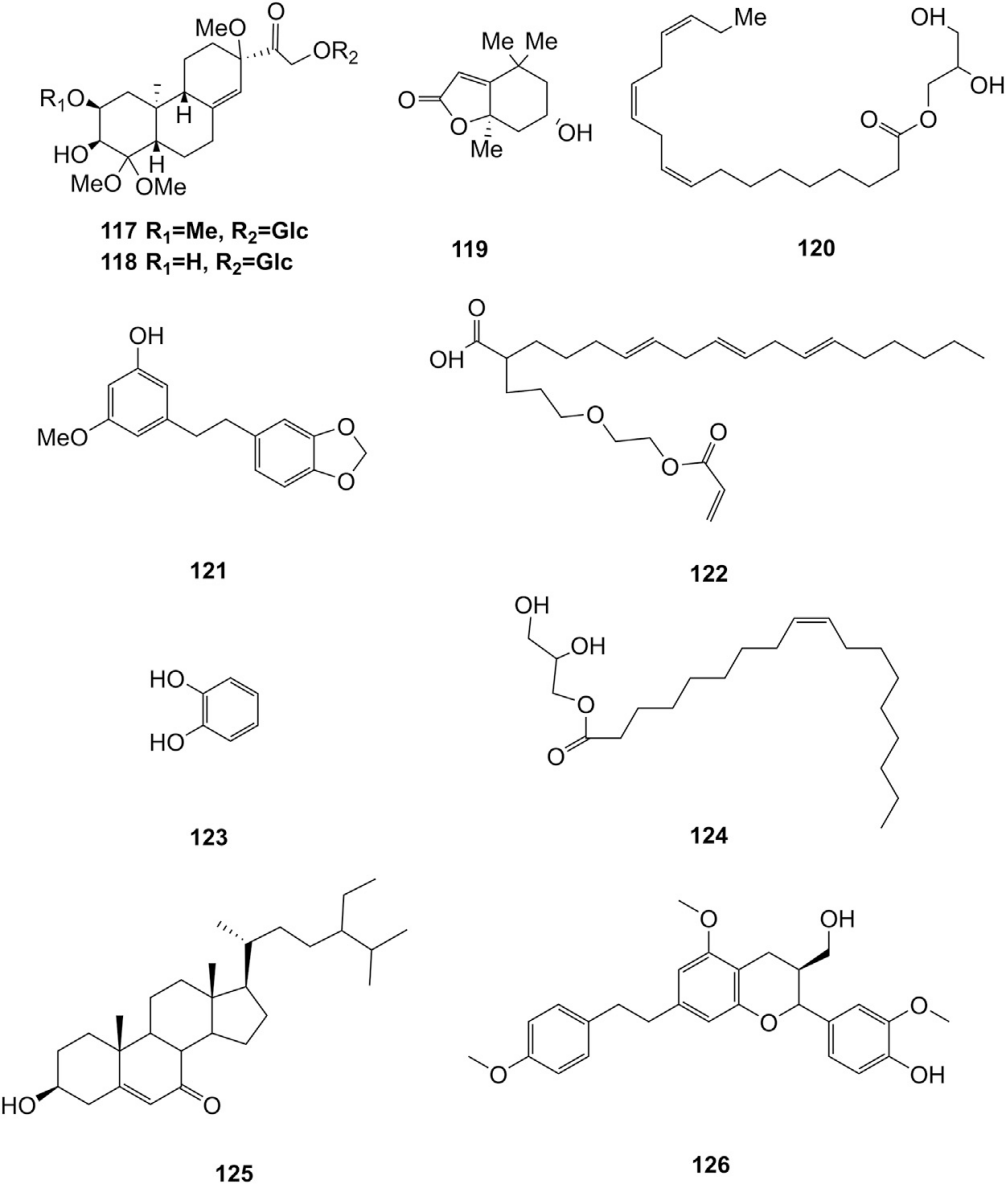
Structures of other compounds (**117–126**) isolated from *D. officinale*.

Some other types of compounds have also been isolated from the stems and leaves of *D. officinale*, including flifimdioside A (**117**), flickinflimoside B (**118**) (Chen et al., 2018), loliolide (**119**), 1-glycerol linolenate (**120**), densiflorol A (**121**), 2-butoxyethyl linolenate (**122**), catechol (**123**) (Ren et al., 2020a), octadecadienoic acid-2,3-dihydroxypropyl ester (**124**) stigmast-5-en-3β-ol-7-one (**125**) (Zhu et al., 2020), and dendrofindlaphenol B (**126**) (Liu et al., 2018). The chemical structures of these compounds are shown in Figure 10.

## Pharmacology

**FIGURE 11:**
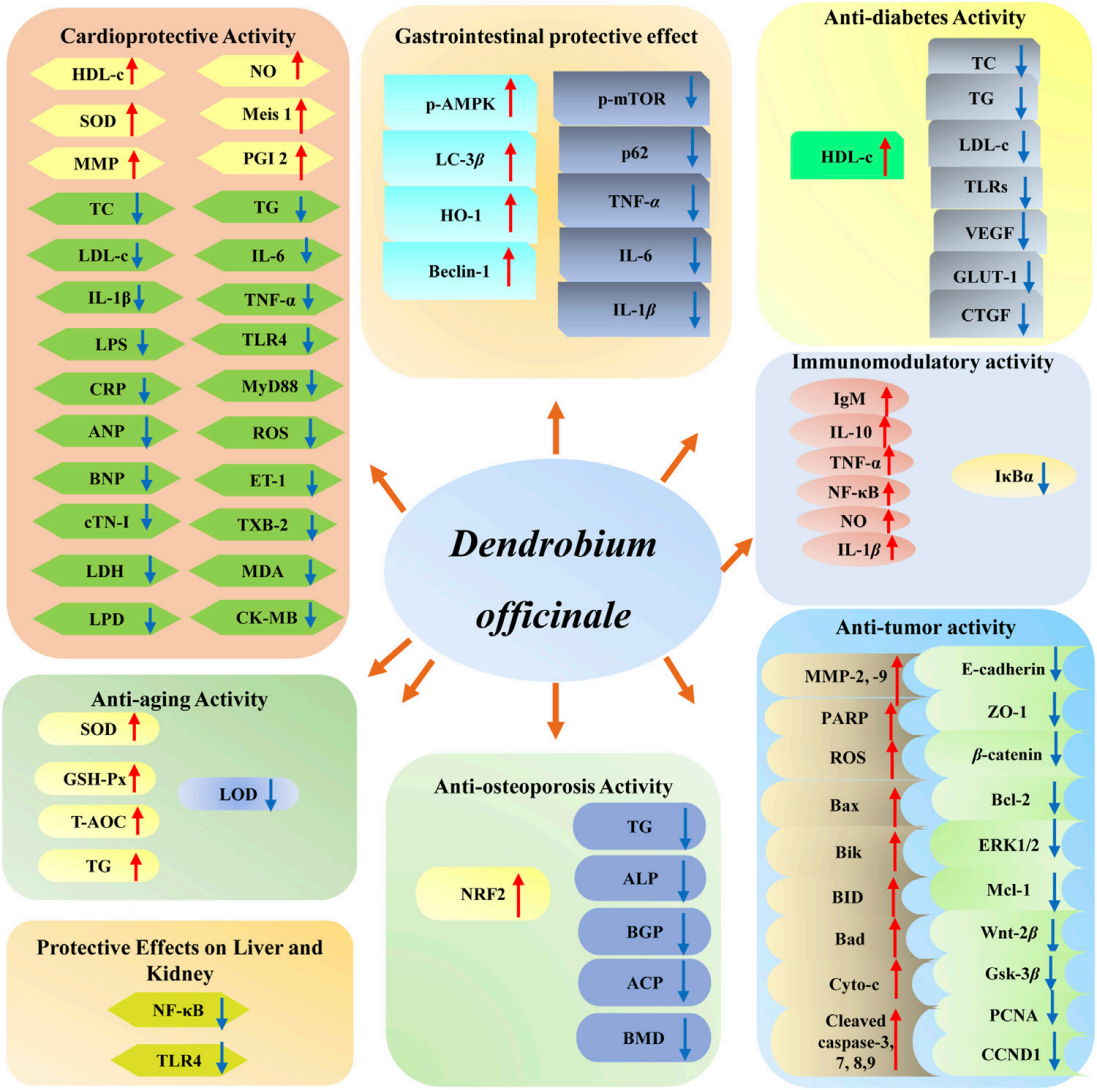
The bioactivities of main compounds isolated from *D. officinale* and their underlying mechanisms.

*D. officinale* has been found to possess multiple biological functions, including cardioprotective (Zhang et al., 2017a; Su et al., 2021), anti-tumor (Guo et al., 2019), gastrointestinal protective (Liu et al., 2020), anti-diabetes (Zeng et al., 2020), immunomodulatory (Huang et al., 2018), anti-aging (Liang et al., 2017), and anti-osteoporosis (Wang et al., 2018) effects. Among them, modern pharmacological studies of *D. officinale* majorly focus on its cardioprotective, anti-tumor, gastrointestinal protective, and hypoglycemic effects. The biological activities of *D. officinale* and corresponding mechanisms are shown in Figure 11.

### Cardioprotective Activity

Cardiovascular diseases are the major causes of mortality globally, and the main cause of more than 10 million people death. Among cardiovascular diseases risk factors, cardiomyopathy and high blood pressure are the most common ones. Oral administration of *D. officinale* fine powders at the doses of 0.09, 0.18, and a very high dose of 1.1 g/kg for 30 days protected isoproterenol (ISO)-induced cardiac hypertrophy, indicated by the decreased myocardial collagen synthesis, increased myocardial fibrosis and ventricular remodeling, and significantly reduced levels of atrial natriuretic peptide (ANP), brain natriuretic peptide (BNP), and cardiac troponin I (cTN-I) in plasma relative to the model group (ISO = 5 mg/kg) (Xiao et al., 2018). In addition, it was well known that diabetic cardiomyopathy was a typical cardiovascular complication mediated *via* hyperglycemia. One study indicated that *D. officinale* water-soluble extracts prevented diabetic cardiomyopathy and might be a candidate for therapeutic use (Zhang et al., 2016). *D. officinale* water extracts (75, 150, and 300 mg/kg) intragastrically once daily for 2 weeks could protect left anterior descending coronary artery (LAD)-induced myocardial ischemia through decreasing creatine kinase (CK)-MB, lactate dehydrogenase (LDH), malondialdehyde (MDA), and increasing superoxide dismutase (SOD) and Meis 1 levels (Dou et al., 2016). Treatment with *D. officinale* stems polysaccharide DOP-GY at the doses of 6.25, 12.5, and 25 µg/ml exhibited protective effects on hydrogen peroxide (H_2_O_2_)-induced H9c2 cardiomyocyte apoptosis *via* phosphatidylinositol/protein kinase B (PI3K)/Akt and mitogen-activited protein kinase (MAPK) signaling pathways, as demonstrated by the decreased levels of LDH, lipid peroxidation damage (LPD), reactive oxygen species (ROS), and pro-apoptosis protein (Zhang et al., 2017a). Another study demonstrated that *D. officinale* polysaccharides could decrease malondialdehyde levels, increase SOD activities, and inhibit the generation of intracellular ROS in H9c2 cardiomyocytes (Zhao et al., 2017). Besides, Yue et al. (2017) obtained a novel homogeneous heteroxylan from alkali-extracted *D. officinale* crude polysaccharide (S32), which possessed significant anti-angiogenic effects (S32, 13.51 μM) on human microvascular endothelial cells (HMEC-1) by inhibiting their migration and disruption of tube formation in a dose-dependent manner, compared with a vehicle group (Yue et al., 2017). However, some of these experimental doses were too high and there was a lack of positive controls.

It has been suggested that *D. officinale* has cardioprotective activity by treating hypertension. It was reported that blood pressure was significantly reduced after treating with *D. officinale* (10 g/d) (Wu et al., 2018b). The treatment of *D. officinale* ultrafine powder (DOFP) at very high doses of 200 and 400 mg/kg for 20 weeks exhibited anti-hypertensive activity on overeating greasy-induced metabolic hypertension in rats by inhibiting the activation of lipopolysaccharide/toll-like receptor 4 (LPS/TLR4) signal pathway, as demonstrated by the decreased levels of total cholesterol (TC), triglyceride (TG), low-density lipoprotein cholesterol (LDL-c), LPS, C-reactive protein (CRP), interleukin 6 (IL-6), TLR4, myeloid differentiation factor (MyD88), IL-1β, and tumor necrosis factor alpha (TNF-α) and the increased levels of high-density lipoprotein cholesterol (HDL-c) and nitric oxide (NO) relative to valsartan (8 mg/kg)-treated positive control (Su et al., 2021). Moreover, Yan et al. (2019a) reported that the effective component of alcohol extract of *D. officinale* in the treatment of metabolic hypertension was apigenin flavonoid glycosides (Yan et al., 2019a). In contrast, more investigations are still needed to reveal the underlying mechanism of the anti-hypertensive effect of apigenin flavonoid glycosides. The results showed that treatment with *D. officinale* flowers at a very high dose of 3.1 g/kg for 6 weeks could significantly improve vascular diastolic function by reducing systolic blood pressure and mean arterial pressure in high glucose and fat compound alcohol-induced hypertensive rats, inhibiting the thickening of thoracic aorta and the loss of endothelial cells, reducing plasma content of endothelin 1 (ET-1) and thromboxane B2 (TXB2), and increasing the content of prostacycline (PGI2) and NO, compared with model control group and valsartan (5.7 mg/kg) positive control group (Liang K. L et al., 2018). Treating with *D. officinale* ultrafine powder DOFP could improve the intestinal flora and increase the production, transportation, and utilization of short-chain fatty acid (SCFA), activate the intestinal-vascular axis SCFA-GPCR43/41 pathway, increase vascular endothelial function, and finally decrease the blood pressure in alcohol, and high sugar and fat diets (ACHSFD)-induced metabolic hypertension model rats (Li et al., 2021). These results suggested that *D. officinale* may have a potential clinical application in the treatment of hypertension. In this study, however, the optimal dose, constituents, and side effects of *D. officinale* are not assessed. Moreover, further detailed clinical trials should be employed to assess the value of *D. officinale* as a drug for the treatment of hypertension. In addition, this evidence is still tenuous; no double-blind trials involving *D. officinale* have been performed, and more evidence from randomized controlled trials is required to elucidate other mechanisms that may be responsible for anti-hypertension effects.

**FIGURE 12:**
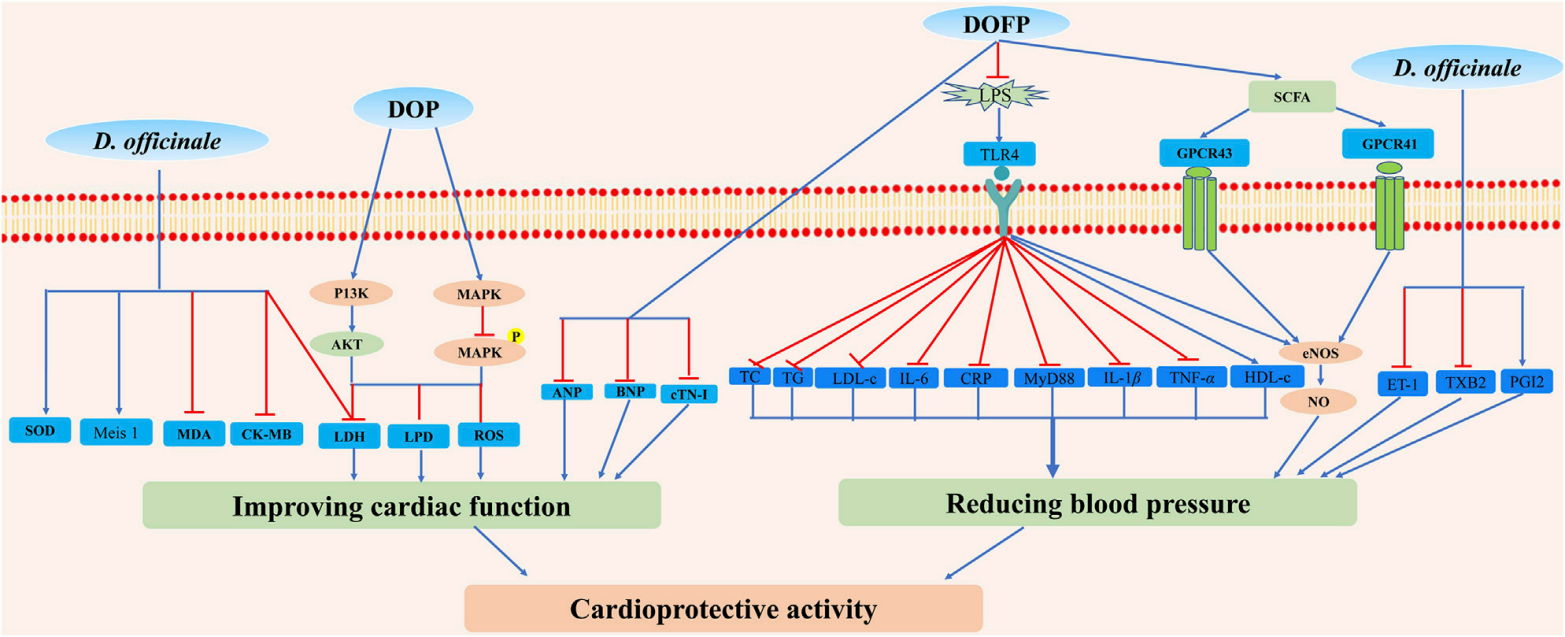
Possible mechanisms for cardioprotective activity properties of *D. officinale*. *D. officinale* exhibited cardioprotective activity by improving cardiac function *via* inhibiting oxidative stress, inflammation, and by reducing blood pressure *via* inhibiting the activation of LPS/TLR4 signal pathway, decreasing ET-1 and TXB2 levels, and increasing SCFA and PGI2 levels.

Although *D. officinale* possesses a potential therapeutic effect on cardiovascular diseases, especially cardiomyopathy and hypertension (Figure 12), more in-depth investigations on its effective monomer compounds, molecular mechanism, and clinical trials are warranted to identify effective cardioprotective agents with minimized side effects.

### Anti-Tumor Activity

As a type of traditional medicine and ordinary food, *D. officinale* has benefits on human health supported by its effectiveness in the prevention and treatment of cancer diseases (Guo et al., 2019). Several studies have reported all crude extracts, polysaccharides, and other pure compounds isolated from *D. officinale* exhibited anti-tumor activities (Wei et al., 2018; Guo et al., 2019; Zhao et al., 2020).

Administration of *D. officinale* methanol extracts at a dose of 0.25, 0.5, and 1 mg/ml could inhibit the growth of SMMC-7721, BEL-7404 cells, and primary liver cancer cells, and promote their apoptosis *via* activating mitochondria apoptosis pathway and suppressing the Wnt/β-catenin pathway (Guo et al., 2019). Similarly, another study demonstrated that *D. officinale* polysaccharide (DOPA-1) induced HepG-2 cell apoptosis by influencing mitochondrial function, ROS production, and apoptosis-related protein expression (Wei et al., 2018). Importantly, a rat study also confirmed the anti-tumor effects of *D. officinale in vivo*. The 2-weeks administration of *D. officinale* polysaccharide DOP at very doses of 2.4, 4.8, and 9.6 g/kg suppressed 1-methyl-2-nitro-1-nitrosoguanidine-(MNNG)-induced (150 µg/ml) precancerous lesions of gastric cancer in rats *via* modulating Wnt/β-catenin pathway and altering endogenous serum metabolites (Zhao et al., 2019). In addition to crude extracts and polysaccharides, it is more evident that gigantol (**4**), moscatilin (**5**), erianin (**7**), orchinol (**25**), and isoviolanthin (**71**) isolated from *D. officinale* are also responsible for the anticancer activity of *D. officinale* (Xing et al., 2018; Liu et al., 2019; Lee et al., 2020; Zhao et al., 2020). Gigantol (**4**) was found to repress invasiveness and growth of SW780, 5,637, and T24 human bladder cancer cells by inhibiting the Wnt/epithelial-mesenchymal transition (EMT) signaling (Guo et al., 2019). Likewise, moscatilin (**5**) was demonstrated to induce apoptosis in FaDu human head and neck squamous carcinoma cells (HNSCC) *via* c-Jun N-terminal kinase (JNK) signaling pathway (Lee et al., 2020). The anti-tumor function of erianin (**7**) was investigated by two independent studies. The results suggest that erianin (**7**) induced cell apoptosis through the ERK pathway in nasopharyngeal carcinoma (NPC) (Liu et al., 2019) while suppressing the growth of bladder cancer cells EJ and T24 through JNK pathways with the IC_50_ values of 65.04 and 45.9 nM, respectively (Zhu et al., 2019). Moreover, it was demonstrated that orchinol (**25**) exhibited strong cytotoxic activity in HI-60 and THP-1 cells with the IC_50_ values of 11.96 and 8.92 μM, respectively (Lee et al., 2020). Additionally, isoviolanthin (**71**) was revealed to suppress transforming growth factor (TGF)-β1-induced EMT through the regulation of TGF-β/Smad and PI3K/Akt/mTOR signaling pathways in HepG2 and Bel-7402 hepatocellular carcinoma (HCC) cells (Xing et al., 2018). However, the dose of *D. officinale* polysaccharide DOP was too high in the treatment of gastric cancer, attention should be paid to the possible side-effects in clinical applications.

**FIGURE 13:**
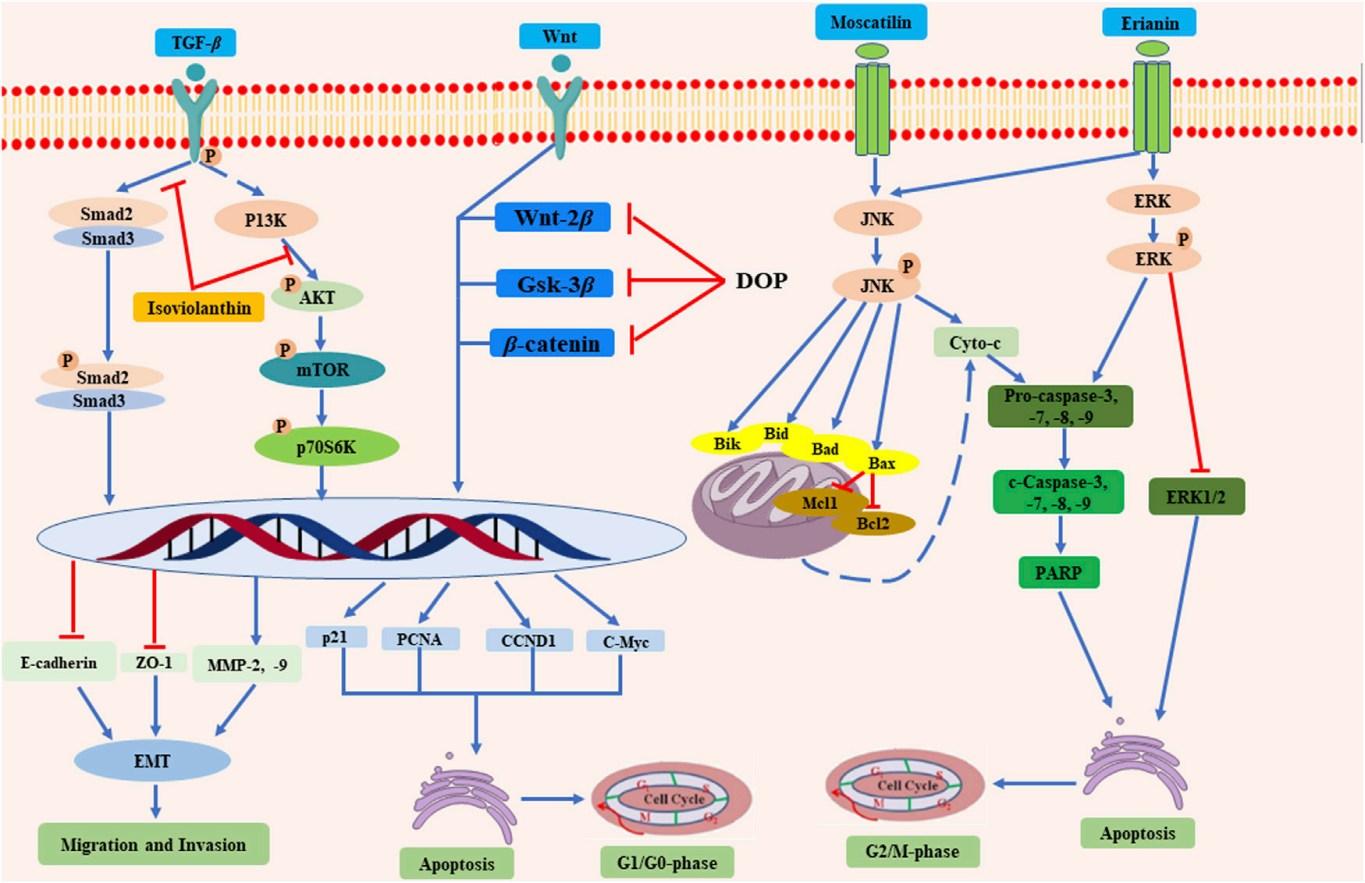
The anti-tumor mechanisms of the compounds from *D. officinale*. *D. officinale* exhibited anti-tumor activity by promoting the tumor cells apoptosis *via* inhibiting the G1/G0 and G2/M phases, and inhibiting the migration and invasion of tumor cells.

Overall, the anti-tumor mechanisms of *D. officinale* are mainly attributed to promoting tumor cell apoptosis, inhibiting tumor cell proliferation, repressing tumor cell migration and invasion, and improving body immunity (Figure 13). The studies described above suggest that the compounds identified in *D. officinale* demonstrate significant inhibitory effects on different types of tumor cells, such as SMMC-7721, BEL-7404, HepG-2, SW780, and HCC cell lines. Further investigations may explore the structural modification and the structure-activity relationship (SAR) studies for these bioactive compounds of *D. officinale*, thereby facilitating the further discovery and development of new anti-tumor candidates, along with the characterization of the key regulatory genes, metabolic pathways, and heterologous biosynthesis pathways of active ingredients from *D. officinale*. The currently published studies on the anti-tumor effects of the compounds from *D. officinale* are mainly focused on *in vitro* and *in vivo* experiments, while clinical studies have yet to be conducted and the exact molecular mechanisms remain elusive.

### Gastrointestinal Protective Effect

*D. officinale* is traditionally used to nourish “Yin” and thicken stomach. *D. officinale* extracts have thus been frequently applied to treat gastrointestinal diseases as a traditional Chinese medicine. It was reported that polyphenols from fermentation liquid of *D. officinale* improved intestinal health *via* the regulation of intestinal microbiota and their metabolites, thereby relieving oxazolone-induced intestinal inflammation in Zebrafish (Gong et al., 2020). Moreover, *D. officinale* is often used in combination with other traditional Chinese medicines to achieve its better therapeutic effects. It was found that the mixture of *D. officinale* and American ginseng at a dose of (0.32 × the dog’s weight × 6 g)/12 kg) could function as a prebiotic agent to enhance SCFA-producing genera and reverse gut dysbiosis (Liu et al., 2020). Likewise, the combination of *D. officinale* and other Chinese herbal medicine, such as *Acanthopanax senticosus*, *Panaxnotoginseng*, *Didymocarpus hancei* and *Valeriana officinalis* can also alleviate gastric mucosal injury (Guo, 2020b).

The 14-days treatment of *D. officinale* glucomannans at 0.16 g/kg produced more SCFAs (mainly acetate and butyrate) in cecum and colon (Shi et al., 2020). It should be noted that polysaccharides LDOP-1 isolated from *D. officinale* could protect ethanol-induced gastric mucosal injury *in vitro* (250, 125, and 62.5 mg/ml for 2 h) and *in vivo* (100 and a very high dose of 400 mg/kg for 30 days) by regulating AMP-activated protein kinase (AMPK)/mTOR signaling pathway demonstrated by the increased levels of p-AMPK, light chain 3β (LC-3β), heme oxygenase-1 (HO-1) and Beclin-1, the decreased levels of p-mTOR and p62, and the reversed levels of caspase3, Bax, and Bcl-2 detected both *in vitro* and *in vivo* (Ke et al., 2020). Furthermore, it was suggested that *D. officinale* polysaccharide (200 mg/kg/d) exhibited protective effects against DSS-induced colitis by inhibiting pro-inflammatory cytokines TNF-α, IL-6, and IL-1β in the colonic mucosa, modulating the abundance of gut microbiota, and promoting the production and utilization of SCFAs in the colon (Zhang et al., 2020). However, positive control or dose-dependent effect analysis was not performed, which might require further validation. In addition, the structure of the correlation between polysaccharide administration and health outcomes, as well as the functional role of the polysaccharides themselves has not been examined in depth.

### Anti-Diabetes Activity

Diabetes is a common disease with glucose metabolism disorder, which seriously affects human health around the world (Ye et al., 2017). Numerous ethnomedicinal studies supported the traditional use of *D. officinale* for clearing heat, nourishing “Yin”, benefiting the stomach, and promoting body fluid, thereby leading *D. officinale* to become an essential medicine to treat “Xiao Ke” disease (Diabetes) for thousands of years (Yan et al., 2019b).

Over the past few years, the hypoglycemic effect of *D. officinale* has become an attractive research field. One study has confirmed that the administration of water extracts from the stems of *D. officinale* at the doses of 75, 150, and a very high dose of 300 mg/kg for 12 weeks decreased serum insulin, TC, TG, LDL-c, and increase HDL-c in high-fat diet**/**streptozotocin (HFD/STZ) (30 mg/kg)-induced diabetic mice in a dose-dependent manner (Zeng et al., 2020). The possible mechanism of *D. officinale* water extracts can be associated with improving lipid transport and suppressing insulin resistance and fibrosis *via* EMT. Another study similarly found that a 4-weeks treatment with *D. officinale* water extracts at very high doses of 350 and 700 mg/kg elevated the liver glycogen synthesis, energy, and amino acid metabolism as well as taurine-mediated defense against oxidative stress in STZ-induced diabetic mice (Zheng et al., 2017). It was found that the treatment of *D. officinale* stem’s water extracts at very high doses of 10 and 20 g/kg for 4 weeks ameliorated insulin resistance by decreasing toll-like receptors (TLRs) and inflammatory response in STZ-induced diabetic rats in comparison with the treatment of dimethyl biguanide (DMBG, 0.0042 g/kg) as a positive control (Zhao and Han 2018b). Moreover, it was reported that the administration of *D. officinale* at the doses of 0.2, 0.4, and 0.8 g/kg for 8 weeks protected against diabetic kidney lesions in STZ-induced (60 mg/kg) rats *via* suppressing vascular endothelial growth factor (VEGF), glucose transporter 1 (GLUT-1), and connective tissue growth factor (CTGF) relative to the irbesartan (17.5 mg/kg)-treated positive control (Chang et al., 2019). Additionally, a previous study also revealed that the hypoglycemic effect of the 3-months combined treatment of *D. officinale* (10 g/d) and metformin (0.5 g/d) was superior to the single-use (Wu et al., 2017). However, these studies have important limitations—only one or two doses of *D. officinale* are used in these studies. In addition, the evaluation of *D. officinale* doses that are much higher, and it has no translational value from a therapeutic viewpoint.

It is evident that the hypoglycemic effect of *D. officinale* is closely related to the inhibition of α-glucosidase and α-amylase (Zhu et al., 2020). Notably, 3,4′-dihydroxy-5-methoxybibenzyl (**2**) and dihydroresveratrol (**12**) isolated from *D. officinale* extracts have been reported to exhibit hypoglycemic activity in an α-glucosidase inhibitory assay with IC_50_ values of 36.05 ± 0.67 and 159.59 ± 0.86 µM, respectively, relative to the acarbose as a positive control (IC_50_ = 152.86 ± 1.43 µM) (Liu et al., 2018). Likewise, 3,4-dihydroxy-4′,5-dimethoxybibenzyl (**3**), 3,4,4′-trihydroxy-5-methoxybibenzyl (**8**), dendrocandin U (**15**), *N*-*p*-coumaroyltyramine (**107**), and *N-trans*-*p*-feruloyltyramine (**108**) have been identified as α-glucosidase inhibitors with IC_50_ values of 199.5 μM, 9.46 *m*M, 403.4, 0.4 and 234.1 μM, respectively, compared with acarbose (IC_50_ = 763.5 μM). Furthermore, the inhibitory effect of 3,4-dihydroxy-4′,5-dimethoxybibenzyl (**3**) on α-amylase (an IC_50_ value of 5.99 mM) was weaker than acarbose (IC_50_ = 0.12 μM) (Chu et al., 2019). Several studies have demonstrated the definite hypoglycemic effect of *D. officinale*, while further investigations are required to identify the specific bioactive components responsible for this activity and clarify the hypoglycemic mechanism of *D. officinale*.

### Immunomodulatory Activity

A large number of experiments have provided evidence for the immunomodulatory activity of *D. officinale*. Polysaccharide is the main active component for immunoregulation. Oral administration of 0.25% *D. officinale* polysaccharide DOW-5B (w/v) for 25 days displayed significant immunomodulatory eﬀects *via* increasing the content of butyrate, immunoglobulin M (IgM), IL-10, and TNF-α *in vivo* (Li et al., 2020a). Another study also demonstrated that *D. officinale* polysaccharide DOP-1-1 stimulated immunity by enhancing the level of nuclear factor kappa-B (NF-κB) while inhibiting the level of IκBα through TLR4 signaling (Huang et al., 2018). Moreover, it was found that *D. officinale* polysaccharides (FDP-1) treatments, ranging from 12.5 to 200 µg/ml, exhibited immunomodulatory activity through increasing cell proliferation and NO and IL-1β production in a dose-dependent manner (Tian et al., 2019). Neither positive control nor dose-effect analysis *in vivo* was assessed in these studies. In addition, there is a general lack of systematic research on the relationship between immune activity and structure of *D. officinale* polysaccharides.

### Anti-Aging Activity

The oral administration with a very high dose of 1 g/kg of *D. officinale* juice and a very high dose of 0.32 g/kg of *D. officinale* polysaccharide for 9 weeks exhibited an anti-aging effect in D-galactose-induced (0.125 g/kg) aging mice, supported by the significantly increased contents of SOD, glutathione peroxidase (GSH-Px) and total antioxidant capacity (T-AOC) in serum, as well as the enhanced SOD level in heart, liver, kidney, and cerebrum (Liang et al., 2017). Similar to this study, the treatment with *D. officinale* polysaccharide (DOP) at the doses of 50 and 100 mg/kg for 4 weeks exhibited more potent anti-fatigue activity than *Rhodiola rosea* extract as a positive control in BALB/c mice, as revealed by the increased TG (or fat) mobilization and the decreased lipid oxidation (LOD) and cell variability of T and B lymphocytes in the weight-loaded swimming test (Wei et al., 2017). However, the underlying mechanisms by which *D. officinale*’s anti-aging effects remain unclear. Additionally, the current pharmacological research lacks component analysis and clinical pharmacological experiments.

### Anti-Osteoporosis Activity

A previous study revealed that the administration of *D. officinale* water extracts at very high doses of 150, 300, and 600 mg/kg for 13 weeks prevented ovariectomy (OVX)-induced bone loss in Wistar rats by decreasing the levels of TG, alkaline phosphatase (ALP), bone glucose protein (BGP) while increasing acid phosphatase (ACP) and bone mineral density (BMD) in comparison with Xian-Ling-Gu-Bao capsule (240 mg/kg) as a positive control (Wang et al., 2018). Meanwhile, *D. officinale* water extract treatments (10, 40, 80 μg/ml) were found to suppress receptor activator expression of the nuclear factor-κB ligand (RANKL)-induced osteoclastogenesis in RAW264.7 cells (Wang et al., 2018). In addition, *D. officinale* polysaccharide DOP treatments at very high doses of 200 or 400 µg/ml exhibited an anti-osteoporosis effect through the activation of NRF2 signaling, thereby attenuating adipogenic differentiation and promoting osteogenic differentiation in BMSCs (Peng et al., 2019). Notably, no reports about the anti-osteoporosis effect of small molecule compounds from *D. officinale* have been documented so far.

### Protective Effects on Liver and Kidney

One study has demonstrated that the treatment with *D. officinale* ethanol extracts at the doses of 4.375 and 17.5 mg/kg for 9 weeks prevented liver and kidney damage in hyperuricemic rats by suppressing the protein levels of NF-κB and TLR4 compared with the model group (0.15% adenine, 10% yeast extract, and 89.85% standard diet) (Lou et al., 2020). Likewise, another study revealed that *D. officinale* flower water extracts (50, 100, 150, and 200 mg/kg) showed protective effects on alcohol-induced (10 ml/kg) liver injury by its anti-steatosis, anti-oxidative, and anti-inflammatory effects (Wu et al., 2020). However, additional evidence from randomized controlled trials is required to identify other regulatory mechanisms that may be responsible for the protective effects on liver and kidney. The bioactive constituents of these extracts also remain unknown.

### Other Activities

In addition to the bioactivities described above, *D. officinale* was also found to have other therapeutic effects, such as neuroprotective effect, anti-photoaging effect, and pulmonary protective function. For instance, in hypoxic-ischemic brain damage (HIBD) neonatal rat model (vehicle group, normal saline = 10 ml/kg), the administration of aqueous extracts of *D. officinale* at the doses of 75, 150, and 300 mg/kg for 14 days suppressed the neuronal apoptosis by reducing cleaved caspase-3 and Bax, increasing Bcl-2, enhancing the expression of neurotrophic factors and K + -Cl—cotransporter 2 (KCC2), and decreasing the expression of hypoxia-inducible factor-1α (HIF-1α) and histone deacetylase 1 (HDAC1), leading to neuroprotective effects in neonatal rats against HIBD (Li and Hong, 2020b). *D. officinale* protocorm treatments at the doses of 10, 25, and 50 mg/ml exerted an anti-photoaging effect through decreasing erythema and protected skin from dryness by increasing CAT, SOD, and GSH-Px expression levels and decreasing thiobarbituric acid reactive substances (TBARS) and MMPs levels relative to the model group (UV irradiation) and positive control group (UV irradiation and a formulation of matrixyl) (Mai et al., 2019). In addition, *D. officinale* polysaccharides prevented lung injury by ameliorating cigarette smoke-induced mucus hypersecretion and viscosity by decreasing the expression of mucin-5AC (MUC5AC) mRNA and secretory protein *in vitro* and *in vivo* (Chen et al., 2020).

## Quality Control

The wild resources of *D. officinale* have gradually decreased, while the supply of artificially cultivated *D. officinale* has increased correspondingly. Consequently, wild resource collection has progressively become a non-mainstream, and the artificial cultivation mode occupies a dominant position in *D. officinale* industry (Ni et al., 2018). According to the description in the Editorial Board of Chinese Pharmacopoeia (2020) edition, the peak area ratio of mannose to glucose, moisture content, total ash content, ethanol extract, and polysaccharide content should reach 2.4–8.0%, less than 12.0%, more than 6.0, 6.5, and 25.0% of *D. officinale* stem, respectively (China Pharmacopoeia Committee, 2020). However, the quality of *D. officinale* may be affected by regions, tissues, harvest time, cultivation techniques, growth years, endophytes, and others which may disturb the long growth cycle (Cheng J et al., 2019). For instance, Li et al. (2017a) found that the contents of polysaccharides in *D. officinale* from Yunnan, Fujian, Jiangsu, and Zhejiang were variable (Li et al., 2017b). Among them, the highest content of polysaccharides was 54.42% in Zhejiang, followed by 43.26% in Fujian (Li et al., 2017a). Previous studies have revealed that the contents of polysaccharides in stems, leaves, and flowers of *D. officinale* are 34.61%, 23.51%, and 13.47%, respectively (Cao et al., 2018). It was also found that the polysaccharide content of *D. officinale* is gradually increased during the entire flower-opening process from buds to full bloom, in which the polysaccharide content is the highest in the full bloom stage (13.75%), followed by the micro bloom stage (11.52%). In contrast, the lowest content appears in the bud stage (9.50%) (Huang et al., 2017). In addition, *D. officinale* is usually planted on trees (fixed to the trunk with fine twine). Therefore, the polysaccharide content of *D. officinale* is significantly affected by different auxiliary tree species. Those planted on the evergreen tree *Phoebe zhennan* as the accessory hosts exhibit the highest polysaccharide content (37.8%) substantially different from those grown on the *Michelia ilsonii*, *Davidia nvolucrate* and *Taxus chinensis* var. *mairei* (Gu and Xie, 2021). Moreover, the polysaccharide content of 5-year-old *D. officinale* stems is the highest, followed by 3-year-old, 4-year-old, 2-year-old, and 1-year-old ones (Qin et al., 2017). Besides, endophytes play an important role in promoting the accumulation of polysaccharides. For example, DO14 (*Pestalotiopsis* sp.) isolated from *D. officinale* treated with 240 mg/L protein-polysaccharide fractions (PPF) can promote the accumulation of polysaccharides. This endophytic fungus could be used as biological fertilizer to improve the yield and quality of *D. officinale* (Zhu et al., 2018). In addition, exploiting suitable artificial-sheltered cultivation mode, screening the best cultivation substrate, and developing aseptic germination technology could be utilized to enhance the quality production of *D. officinale* in current agronomical practices (Cheng Y et al., 2019; Zuo et al., 2020).

It is well known that the polysaccharides’ bioactivities vary from different sources, production regions, and cultivation conditions of *D. officinale*. Moreover, it has been reported that the bioactivities of *D. officinale* polysaccharides are related to their chemical characteristics and advanced structures. However, *D. officinale* polysaccharides are macromolecular compounds with large molecular weight and complex structure, thereby generating a great challenge to implement analytical technologies (Ma et al., 2018). Therefore, it is necessary to establish a safe and effective quality assessment method for the quality control and clinical application of *D. officinale*. Herein, the qualitative and quantitative analytic methods of *D. officinale* polysaccharides are summarized and discussed.

There are several rapid and accurate methods for quantitative estimation of natural polysaccharides and their different fractions in *D. officinale* (Wu et al., 2018a). Generally, the large *D. officinale* polysaccharides are acid hydrolyzed into oligosaccharides, and then LC-MS is used to separate and characterize the products efficiently. The previous results indicate that the variations in the mass values of different peaks present structural differences of various metabolized products. Moreover, MS can be used to explore the oligosaccharide hydrolysates of *D. officinale* polysaccharides in detail from the aspects of identity, structure, and properties (Ma et al., 2018). An oligosaccharide-marker approach by labeling them with fluorescence reagent paminobenzoic acid ethyl ester (ABEE) (Te-Man-ABEE and Pen-Man-ABEE) was recently applied for quality assessment of *D. officinale* polysaccharides using UHPLC-QTOF-MS (Wong et al., 2019). The results revealed that the two oligosaccharide markers exhibited a satisfactory linearity relationship with *D. officinale* polysaccharides (R^2^ ≥ 0.997) in the range of 0.68–16.02 µg. These markers also revealed satisfactory precision (relative standard deviation, RSD <7.0%) and recovery (91.41–118.30%) in unknown sample determination. It is speculated that the oligosaccharide-marker method is a simple, rapid, and reliable approach for the qualitative and quantitative determination of specific polysaccharides from *D. officinale* and other herb formulas (Wong et al., 2019).

Although single-component quality assessment can be used to control the quality of natural Chinese herbal medicine (Wei et al., 2020), the chemical compositions of *D. officinale* are complex. *D. officinale* contains a lot of effective nutritional compositions, including flavonoids, crude fiber, amino acids, proteins, and fat. These active components of *D. officinale* are affected by tissues and harvest times. A previous study found that the contents of flavonoids in the stems, leaves, and flowers of *D. officinale* were 0.052%, 0.251%, and 1.835%, respectively (Li et al., 2019a). Another research reported that the content of total flavonoids in the flowers of *D. officinale* is the highest at the full flowering stage (1.66%) followed by the bract stage (1.52%) and micro flowering stage (1.41%) (Huang et al., 2017). It should be noted that the contents of five representative flavonoid glucosides from *D. officinale* in 25 batches with different sources were determined by UHPLC-ESI-MS/MS, where principal component analysis (PCA) and hierarchical cluster analysis (HCA) were applied (Ye et al., 2017). The content of crude fiber in the autumn stem is 59.7% higher than that in the spring stem, and the fiber in the autumn leaf is 122.9% higher than that in the spring leaf (Li et al., 2017a). The total protein content of *D. officinale* stems in autumn is 10.9% higher than that in spring, while that of the leaves in autumn is 9.3% higher than that in spring. The results also revealed that the total protein content in the leaves is higher than that in the stems, and the ratio of total protein in the autumn leaves is 67.03 mg/g, which is 31.4% higher than that in the autumn stems (51.02 mg/g). The content of fat in the stems and leaves of *D. officinale* ranges from 10.0 to 15.0 mg/g. The average content of fat in the leaves (13.90 mg/g) is slightly higher than that in the stems (11.50 mg/g) (Liao et al., 2018). In particular, Wu and Feng (2019) revealed that the content of polysaccharides is the highest in those samples collected from October to the following March (Wu and Feng, 2019). Yu et al. (2014) found that it is more reasonable to harvest *D. officinale* at biennials pre-bloom than at specific harvesting months according to the content of polysaccharides (Xu et al., 2021). According to the Editorial Board of Chinese Pharmacopoeia (2020) edition, however, *D. officinale* should be harvested from November to the following March (China Pharmacopoeia Committee, 2020).

Additionally, there are some noteworthy scientific gaps, which can be resolved from the following aspects. First, the genuine, defective, and counterfeit varieties of *D. officinale* are mixed together, so there is an urgent need to breed varieties with better agronomic characters, high yield, high quality, and intense stress resistance. (Guo et al., 2019). Second, it is also an effective way to ensure the quality of *D. officinale* by controlling pesticide residues and using endophytes to control diseases and pests (Yu et al., 2014; Zhou et al., 2018). Third, at present, chemical methods are primarily used to evaluate the quality of *D. officinale*, while the quality evaluation of traditional Chinese medicine is closely related to its biological activity. Therefore, the evaluation methods are suggested to be improved by combining chemical approaches and bioassays, providing a foundation for the industrial production and clinic use of *D. officinale* (Wei et al., 2020).

## Safety

The stems and leaves of *D. officinale* were approved by National Health and Family Planning Commission (NHFPC) of People’s Republic (PR) of China to be utilized as a novel food material on Jan 15, 2013 and Jan 15, 2017 (National Health and Family Planning Commission of the People’s Republic of China, 2013; National Health and Family Planning Commission of the People’s Republic of China, 2017), respectively. Therefore, several safety studies of *D. officinale* have been performed. It has been reported that acute toxicity test (12.0 g/kg), genetic toxicity tests (Ames test, micronucleus test of bone marrow, and sperm shape abnormality test in mice) (1,000, 2000, and 4,000 mg/kg), and 90-days feeding test (1.08, 1.67, and 5.00 g/kg) in rats were employed to assess the safety of the stems from *D. officinale*. These results indicated that *D. officinale* was a type of health food product without noticeable toxicity, genetic toxicity, and mutagenicity within the range of the test doses (Li Z. et al., 2019). Moreover, oral administration with *D. officinale* stems at the doses of 25, 1,250, and 2,500 mg/kg did not exhibit any apparent effect on pregnant rats or deformity effect on fetal rats (Qin et al., 2019). Likewise, oral administration with the leaves and flowers of *D. officinale* at the doses of 0, 2.0, 4.0, and 6.4 g/kg for 90 days did not exhibit apparent adverse effects on sperm quality and testicular tissue morphology in parent and offspring rats (Fu et al., 2017; Fu et al., 2020a). Besides, *D. officinale* flowers (0, 2.0, 6.4 g/kg) had no apparent adverse effects on pregnant and offspring rats before birth (Fu et al., 2020b).

*D. officinale* is considered to have edible and medicinal values. However, due to its thermal tonic property, it is prohibited for patients with wind-heat cold, dampness, and allergies, teenagers, and pregnant women. According to the Pharmacopoeia of the People’s Republic of China (China Pharmacopoeia Committee, 2020), the dosage of 6–12 g/d of *D. officinale* is appropriate, and intake of trace elements recommended by the Food and Drug Administration of the United States will not induce poisonous effects.

## Conclusion and Future Prospects

*D. officinale*, as a medicinal or food homologous product, plays a crucial role in healthcare. This study summarizes and updates the botany, traditional uses, bioactive components, pharmacology, quality control, and safety of *D. officinale*. Available data indicate that over 120 compounds have been isolated and identified from *D. officinale*, including polysaccharides, bibenzyls, flavonoids, alkaloids, phenanthrenes, etc. *D. officinale* is associated with multiple beneficial pharmacological properties, such as cardioprotective, anti-tumor, gastrointestinal protective, anti-diabetes, anti-aging, and anti-osteoporosis activities. Furthermore, it is evident that *D. officinale* is a non-toxic, which can be listed as a toxicologically safe functional food. However, its clinical applications has been rarely described, and critical improvements are still required for its industrial applications.

Firstly, phytochemical studies have demonstrated that *D. officinale* mainly contains polysaccharides, bibenzyls, phenanthrenes, and flavonoids while little is known about the analysis and function of organic compounds such as protein and fatty acid. Growing evidence has shown that structure-based drug design plays a vital role in developing novel drugs, and a series of strategies can be adopted to obtain effective therapeutic drugs. Further studies need to be conducted to isolate and identify more compounds from *D. officinale* with novel structures, emphasizing on bioactivity-guided, structurally modified, and chemically synthesized molecules. Besides, most of the *D. officinale* containing health products are mainly derived from its stems rich in chemical compounds, while non-medicinal parts are rarely exploited. Therefore, it may be interesting to extend the research to the non-medicinal parts of the inexpensive flowers, leaves, and roots of *D. officinale* to ensure the fully utilization of its edible and medicinal values (Zhang et al., 2017a).

Second, *D. officinale* is traditionally used to relieve fatigue, nourish “Yin”, heart, and stomach, and expel evil heat, which is closely related to its preventive effects on cardiac, gastrointestinal and diabetes diseases according to modern pharmacology. Pharmacological research of the cardioprotective, gastrointestinal tract and anti-diabetes protective effects of *D. officinale* mainly concentrates on its crude extracts and polysaccharides. However, the optimal dose, constituents, and side effects of *D. officinale* are not assessed. Besides, there is a lack of in-depth study on the mechanism of action of *D. officinale* monomeric compounds or the specific mechanism of *D. officinale* in animals is not comprehensive enough. Therefore, the high-quality and well-designed *in vivo*, *in vitro*, and clinical studies are encouraged to be carried out and to explore the molecular mechanisms and relationship between active chemical constituents and potential cardioprotective, gastrointestinal tract regulatory and anti-diabetes effects.

Third, the dosage of *D. officinale* used in previous studies were different (ranging from 4.375 mg/kg to 20 g/kg). It is not possible to define an exact upper cut-off dose, and the test dose needs to be pharmacologically relevant. In many cases, 100–200 mg/kg extracts for *in vivo* studies should be assumed as the upper limit for meaningful pharmacological studies. For pure compounds, a much lower dose range (e.g., 30–50 μM) should be considered for *in vitro* studies (Heinrich et al., 2020). In some cases, the pharmacological activity of *D. officinale* is present only with doses that might be too high for clinical use. According to the Chinese Pharmacopoeia, 6–12 g/day administration of *D. officinale* extract is common for human, but any clinical application at such doses should be accompanied with conservative safety insurance. Besides, the acute toxicity and sub-toxicity assessments of *D. officinale* were mostly carried out based on animal experiments. Furthermore, comprehensive placebo-controlled and double-blind clinical trials are necessary to provide sufficient evidence ensuring drug efficacy and patient safety.

Finally, *D. officinale* possesses various biological activities, which has been applied as health care medicine, health food, and health tea. In addition, with the development of analytical techniques and quality control methods, such as the improvement and update in chromatography techniques and molecular identification methods, new quality markers and quality control measures are likely to be adopted for better quality assessment of Chinese herbal medicine in the future (Leong et al., 2020).

In conclusion, *D. officinale* is one of the most popular medicinal and food homologous products in China. Modern pharmacology investigations have revealed its cardio-protective, gastrointestinal protective, anti-diabetes, and anti-aging effects, which robustly support its traditional application in nourishing “Yin”, heart, and stomach, expelling evil heat, and relieving fatigue. This paper provides a full-scale review about the progress of botany, traditional uses, phytochemistry, pharmacology, quality control, and toxicology of *D. officinale*. The information summarized in this work can provide a foundation for further applying the medicinal and edible value of *D. officinale* in the future.

## Glossary

D. officinale: Dendrobium officinale
HPLC: high performance liquid chromatography
HPAEC: high performance gel permeation chromatography
GC-MS: gas chromatography-mass spectrometry
GPC: gel permeation chromatography
HPLC-ESI-MS: high performance liquid chromatography electrospray ionization mass spectrometry
UHPLC: ultra-high performance liquid chromatography
QTOF: quadrupole time of flight mass spectrometry
ISO: isoproterenol
ANP: atrial natriuretic peptide
BNP: brain natriuretic peptide
cTN-I: cardiac troponin I
LAD: anterior descending coronary artery
CK: creatine kinase
LDH: lactate dehydrogenase
MDA: malondialdehyde
SOD: superoxide dismutase
H2O2: hydrogen peroxide
PI3K: phosphatidylinositol
Akt: protein kinase B
MAPK: mitogen-activited protein kinase
LPD: lipid peroxidation damage
ROS: reactive oxygen species
HMEC-1: human microvascular endothelial cells
DOFP: D. officinale ultrafine powder
LPS: lipopolysaccharide
TLR4: toll-like receptor 4
TC: total cholesterol
TG: triglyceride
LDL-c: low-density lipoprotein cholesterol
CRP: C-reactive protein
IL-6: interleukin 6
MyD88: myeloid differentiation factor
TNF-α: tumor necrosis factor alpha
HDL-c: high-density lipoprotein cholesterol
NO: nitric oxide
ET-1: endothelin 1
TXB2: thromboxane B2
PGI2: prostacycline
SCFA: short-chain fatty acid
ACHSFD: alcohol, and high sugar and fat diets
MNNG: methyl-2-nitro-1-nitrosoguanidine
EMT: epithelial-mesenchymal transition
HNSCC: human head and neck squamous carcinoma cells
JNK: jun N-terminal kinase
NPC: nasopharyngeal carcinoma
TGF: transforming growth factor
HCC: hepatocellular carcinoma
SAR: structure-activity relationship
AMPK: AMP-activated protein kinase
LC-3β: light chain 3β
HO-1: heme oxygenase-1
HFD/STZ: high-fat diet/streptozotocin
TLRs: toll-like receptors
DMBG: dimethylbiguanide
VEGF: vascular endothelial growth factor
GLUT-1: glucose transporter 1
CTGF: connective tissue growth factor
IgM: immunoglobulin M
NF-κB,: nuclear factor kappa-B
GSH-Px: glutathione peroxidase
T-AOC: total antioxidant capacity
DOP: D. officinale polysaccharide
LOD: lipid oxidation
OVX: ovariectomy
ALP: alkaline phosphatase
BGP: bone glucose protein
ACP: acid phosphatase
BMD: bone mineral density
RANKL: receptor activator expression of the nuclear factor-κB ligand
Nrf2: nuclear factor E2-related factor 2
KCC2: K + -Cl -cotransporter 2
HIF-1α: hypoxia-inducible factor-1α
HDAC1: Histone deacetylase 1
HIBD: hypoxic-ischemic brain damage
TBARS: thiobarbituric acid reactive substances
MUC5AC: mucin-5AC
ABEE: aminobenzoic acid ethyl ester
RSD: relative standard deviation
PCA: principal component analysis
HC: hierarchical cluster analysis
NHFPC: national health and family planning commission
PR: people’s republic
